# High EMT Signature Score of Invasive Non-Small Cell Lung Cancer (NSCLC) Cells Correlates with NFκB Driven Colony-Stimulating Factor 2 (CSF2/GM-CSF) Secretion by Neighboring Stromal Fibroblasts

**DOI:** 10.1371/journal.pone.0124283

**Published:** 2015-04-28

**Authors:** Albin Rudisch, Matthew Richard Dewhurst, Luminita Gabriela Horga, Nina Kramer, Nathalie Harrer, Meng Dong, Heiko van der Kuip, Andreas Wernitznig, Andreas Bernthaler, Helmut Dolznig, Wolfgang Sommergruber

**Affiliations:** 1 Department of Lead Discovery, Boehringer Ingelheim RCV GmbH & Co KG, Vienna, Austria; 2 Department of Microbiology, Immunobiology and Genetics, Center of Molecular Biology, Max F. Perutz Laboratories, University of Vienna, Vienna, Austria; 3 Institute of Medical Genetics, Medical University of Vienna, Vienna, Austria; 4 Dr. Margarete Fischer-Bosch Institute of Clinical Pharmacology and University of Tübingen, Stuttgart, Baden-Württemberg, Germany; Faculdade de Medicina Dentária, Universidade do Porto, PORTUGAL

## Abstract

We established co-cultures of invasive or non-invasive NSCLC cell lines and various types of fibroblasts (FBs) to more precisely characterize the molecular mechanism of tumor-stroma crosstalk in lung cancer. The HGF-MET-ERK1/2-CREB-axis was shown to contribute to the onset of the invasive phenotype of Calu-1 with HGF being secreted by FBs. Differential expression analysis of the respective mono- and co-cultures revealed an upregulation of NFκB-related genes exclusively in co-cultures with Calu-1. Cytokine Array- and ELISA-based characterization of the “cytokine fingerprints” identified CSF2 (GM-CSF), CXCL1, CXCL6, VEGF, IL6, RANTES and IL8 as being specifically upregulated in various co-cultures. Whilst CXCL6 exhibited a strictly FB-type-specific induction profile regardless of the invasiveness of the tumor cell line, CSF2 was only induced in co-cultures of invasive cell lines regardless of the partnered FB type. These cultures revealed a clear link between the induction of CSF2 and the EMT signature of the cancer cell line. The canonical NFκB signaling in FBs, but not in tumor cells, was shown to be responsible for the induced and constitutive CSF2 expression. In addition to CSF2, cytokine IL6, IL8 and IL1B, and chemokine CXCL1 and CXCL6 transcripts were also shown to be increased in co-cultured FBs. In contrast, their induction was not strictly dependent on the invasiveness of the co-cultured tumor cell. In a multi-reporter assay, additional signaling pathways (AP-1, HIF1-α, KLF4, SP-1 and ELK-1) were found to be induced in FBs co-cultured with Calu-1. Most importantly, no difference was observed in the level of inducibility of these six signaling pathways with regard to the type of FBs used. Finally, upon tumor fibroblast interaction the massive induction of chemokines such as CXCL1 and CXCL6 in FBs might be responsible for increased recruitment of a monocytic cell line (THP-1) in a transwell assay.

## Introduction

Worldwide, lung cancer is the leading cause of cancer-related mortality and by 2010 was the fifth overall leading cause of death. Globally, lung cancer attributes approximately 1.37 million deaths per year with non-small cell lung cancer (NSCLC) as the most common form of lung cancer. About two thirds of patients with NSCLC present with advanced disease which allows only limited treatment options [1]. Although standard treatment regimens have achieved promising results with neoadjuvant and adjuvant strategies, outcomes for patients with lung cancer are still considered disappointing. Recent data provide evidence that the tumor-stromal environment is a key player in carcinogenesis. Therefore, genes involved in tumor-stroma interactions may represent novel candidate targets for therapeutic intervention in lung cancer [2].

Carcinomas constitute highly complex structures composed of genetically altered tumor cells, normal fibroblasts (NFs), cancer-associated fibroblasts (CAFs), endothelial cells, pericytes and inflammatory cells all embedded in an extracellular matrix (ECM) of proteins [3]. An array of growth factors and cytokines secreted by the surrounding stromal cells plays a major role in tumorigenesis and metastasis. Notably, cell-to-cell interactions result in the activation of numerous signaling pathways. Among all the stromal cells, fibroblasts (FBs) are essential to synthesize and deposit the ECM by producing a variety of collagen and fibronectin [4]. CAFs actively participate in the growth and invasion of the tumor cells by providing a unique tumor microenvironment [5]. Conversely, NFs can inhibit the proliferation of pre-cancerous breast epithelial cells. This inhibitory capacity of NFs is often reduced, or reversed, in CAFs [6] and can even stimulate the proliferation of epithelial cells. The role that CAFs play in transformation, proliferation and invasion in breast cancer is achieved through the ability to secrete growth factors and chemokines. These secretions lead to critical changes in the ECM and exert oncogenic signals resulting in increased tumor cell proliferation and invasion [7]. Recently, CAFs have been shown to regulate the plasticity of lung cancer stemness via paracrine signaling through CAF-derived IGF-II and IGF1R signaling. This induces the expression of Nanog and thereby promoting stem-cell like characteristics in lung cancer cells. In this way, CAFs constitute a supporting niche for cancer stemness [8]. CAFs are therefore considered not merely a simple physical supporting element of the parenchymal or carcinoma cells but also a functionally important regulatory component of the tumor microenvironment [9]. Autocrine and paracrine interactions between cancer and stromal cells are regarded as pivotal for carcinogenesis and are also being considered as novel targets for therapy. FBs are particularly attractive therapeutic targets due to their genetic stability and reduced heterogeneity compared to cancer cells [10]. In clinical trials, several drugs targeting the microenvironment have been tested including targets such as VEGF and its receptors on NSCLC-associated endothelial cells [2,11] or on reactive FBs in pulmonary fibrosis [12]. The tumor microenvironment may even promote resistance to chemotherapy [13] as shown for example for the stromal cell-derived factor-1 (SDF-1) secreted in the lung microenvironment of lung metastases [14].

A further contribution of CAFs during tumorigenesis is the promotion of the immunologic tolerization of the tumor as a result of imbalances in the tumor microenvironment, including alterations in antigen-presenting-cell subsets, co-stimulatory and co-inhibitory molecule alterations such as CXCR4, IL2 or CCL2 and altered ratios of effector T cells and regulatory T cells [15]. Another example for a tumor-stroma interaction partner is IGFBP7, which was identified as a promoter of anchorage-independent growth in malignant mesenchymal cells and in epithelial cells with an EMT phenotype. IGFBP7 can induce colony formation in colon cancer cells co-cultured with IGFBP7-expressing CAFs by a paracrine tumor-stroma interaction [16]. A number of publications have shown that NFκB signaling plays a major role in tumor-stroma interactions. For instance, NFκB was identified to be upregulated in several inflammation-linked cancers [17] and pro-inflammatory signaling by CAFs in squamous cell carcinoma turned out to be NFκB-dependent [18]. Furthermore, pro-tumorigenic signaling in the microenvironment of breast and ovarian tumors via the NFκB pathway is mediated by CAFs [19].

Despite the availability of numerous data sets describing the essential contribution of inflammatory signaling to tumorigenesis, a detailed understanding of the molecular mechanisms of this crosstalk is still missing. The absence is particularly apparent for lung cancer [1]. In order to more precisely understand the molecular basis of this crosstalk we established several *in vitro* mono- and co-cultures of lung tumor cells and various types of FBs including patient-derived NFs and CAFs to identify specifically induced signaling pathways. In addition, the influence of these pathways on alterations in expression profiles was investigated with a special focus on differential cytokine profiles. Furthermore, we examined the correlation between altered expression profiles and the level of tumor cell invasiveness utilizing different types of FBs. In contrast to the generally accepted opinion that mainly CAFs contribute essentially to the tumor-stroma crosstalk [18,19] we demonstrate that FBs of different origins such as human dermal FBs (HDFs), NFs and CAFs can equally contribute to the crosstalk with lung cancer cells. This particularly holds true for the induction of the cytokine CSF2 (GM-CSF), whose expression is triggered exclusively by lung tumor cells exhibiting a high EMT signature.

## Materials and Methods

### Cell Culture

Cells were cultured in a humidified incubator at 37°C and 5% CO_2_. NCI-H1437 (CRL-5872), A549 (CCL-185), NCI-H460 (HTB-177), NCI-H226 (CRL-5826) cell lines were grown in RPMI1640 + GlutaMAX (RPMI) medium (Gibco, 35050) supplemented with 10% fetal bovine serum (FBS) (Gibco, 10082), Calu-1 (HTB-54) in McCoy’s 5A modified + GlutaMAX medium (Gibco, 36600) supplemented with 10% FBS and NCI-H157 (CRL-5802) in RPMI supplemented with 10% FBS, non-essential amino acids (Gibco, 11140), sodium pyruvate (Gibco, 11360) and 10 mM HEPES (Affymetrix, 16924). All lung cancer cell lines were obtained from the ATCC. Neonatal human dermal fibroblasts (HDFs) were purchased from Innoprot (P10857) and cultured in Fibroblast Growth Medium (Promocell, C-23010). WI-38 (CCL-75) and IMR-90 (CCL-186) normal fetal lung FBs were obtained from the ATCC. Both FB lines were maintained in MEM Eagle Medium (EMEM) (Lonza, BE12) supplemented with 1 mM GlutaMAX-I (Gibco, 35050), 10% FBS. The isogenic primary normal (NF) and cancer-associated FB (CAF) pairs NF1 & CAF1 and NF2 & CAF2 had been isolated as described previously [20] and were cultured in RPMI supplemented with 10% FBS. For further details of all cell lines see S1 Table. The local ethics committee ‘Ethik-Kommission der Medizinischen Fakultät am Universitätsklinikum Tübingen’ approved the investigation (project number 396/2005V and 159/2011BO2) and a written informed consent was obtained from each patient. Experiments using primary FBs were performed at early passages (<P5). For several experiments recombinant human HGF (R&D, 294-HG) was added to the growth medium. Anti-human HGF antibody was from R&D (R&D, MAB294). The MET specific inhibitor crizotinib was purchased from Selleckchem (S1068). All cell cultures were supplemented with Pen/Strep (Gibco, 15140). NCI-H1437 and Calu-1 cells were modified to stably express TurboGFP, using a lentiviral system (Sigma Aldrich, SHC003V). Accordingly, HDF, WI-38 and IMR-90 FBs were transfected to express tagRFP (Sigma Aldrich, SHC012V).

### Cytokine Antibody Arrays and ELISA Assays

For mono-cultures 2x10^6^ tumor or FB cells were seeded in a 100 mm dish (Corning, 35300) with a total volume of 10 ml of RPMI medium supplemented with 10% FBS and Pen/Strep. Co-cultures were prepared with 2x10^6^ cells for each cell type per dish. Medium was exchanged after 24 h. Supernatants were harvested after another 24 h of incubation and stored at -80°C. Cytokine antibody arrays were performed with human cytokine array kits from R&D Systems (ARY005) and RayBiotech (AAH-CYT-1000) following the manufacturer’s protocols. The X-ray films were scanned and individual array spots were analyzed and quantified by using the GenePix Pro 7.0 software. The following ELISA kits were purchased from R&D Systems for cross-validation of identified hits: IL-6 (D6050), IL-8 (D8000C), GCP-2 (DGC00), CXCL1 (DGR00), GM-CSF (DGM00) and VEGF (DVE00), respectively. ELISA assays were performed following the manufacturer’s protocol.

### Reporter Assay

First, 1x10^5^ cells per well were seeded in a white, clear bottom 96-well plate (Perkin Elmer, 6005181) in a final volume of 100 μl per well. On day two cells were transfected with 100 ng reporter plasmids derived from either reporter array plates obtained from Qiagen (CCA-901L-12) or single reporter constructs by using Lipofectamine LTX (Life Technologies, 15338100) supplemented with Plus Reagent. 24 h later plates were washed with 1X PBS and 1x10^5^ cells of the respective co-culture were added to the transfected cells harboring the reporter construct. Trametinib, a MEK1/2 inhibitor (Selleckchem, CAS No. 871700-17-3) or BI5700, a small molecule inhibitor of IκB kinase 2 (IKK2/IKKß) was added optionally. The thienopyridine BI5700 was derived from a chemical lead optimization program designed for selective IKK2 inhibitors (issued patent US6974870). On day four the Luciferase reporter signal was measured with the Dual-Glo Luciferase Assay System (Promega, E2940) and normalized to the corresponding Renilla control signal.

### Transwell Assay

FBs were seeded into the lower chamber of a 24-well plate, whereas tumor cells were plated separately into a transwell insert with 0.4 μm pores (Costar, 3381) and incubated at 37°C overnight. After 24 h the medium was exchanged, the FB containing bottom chambers were overlaid with the pre-incubated tumor cell inserts and incubated for another 24 h. After separation of inserts from the lower chambers, total RNA was isolated and first strand cDNA synthesis was performed using the QiagenRNeasy Mini Kit (Qiagen, 74804) following the manufacturer’s instructions. Transwell migration assays were performed using transwell inserts with 3 μm pores (Costar, 3399). Tumor cells and FBs were seeded in a 24-well plate and incubated overnight at 37°C. After exchanging medium inserts with 3 μm pore size were put on top containing a cell suspension of THP-1 monocytes, which constitutively express a luciferase reporter. After an additional 24 h, the inserts were removed and cells in the bottom chamber were lysed and assayed using the Dual-Glo Luciferase Assay System.

### Quantitative Real-Time PCR (RT-qPCR)

RT-qPCR was performed in a Real-Time PCR Detection System (Applied Biosystems, StepOne Plus Real-Time PCR System) using the TaqMan assay technology (Life Technologies). A total of 12.5 μl of QT MP RT-PCR Mastermix (Qiagen, 74804) was mixed with 1.25 μl of both FAM- (gene of interest) and VIC-labeled (reference gene) TaqMan assays (Life Technologies), 0.25 μl of QT MP RT Mix (Qiagen, 74804) and 9.75 μl of diluted cDNA. Data analysis was performed according to the ΔΔC_t_ method using Hypoxanthin-Guanine-Phosphoribosyltransferase (HPRT) and Beta-2 microglobulin (B2M) as endogenous controls. The following TaqMan assay primers from Applied Biosystems were used: IL6 (Hs00985639_m1), CSF2 (Hs00929873_m1), CXCL1 (Hs00605382_gH), IL8 (Hs00174103_m1), IL1B (Hs01555410_m1) and CXCL6 (Hs00605742_g1).

### Western Blot Analysis

Total cell extracts were fractionated by gel electrophoresis. Proteins were transferred to PVDF membranes and immunoblotted using following antibodies: GAPDH (abcam, ab8245), ß-Actin (abcam, ab8226), Met (L41G3, Cell Signaling, 3148) and Phospho-Met (Tyr1234/1235) (Cell signaling, 3126), MAPK (Erk1/2) (06–182, Millipore) and Phospho-p44/42 MAPK (Erk1/2) (Thr202/Tyr204) (9101, Cell Signaling). The protein-antibody complexes were detected by horseradish peroxidase-conjugated secondary antibodies incubated with a chemiluminescent reagent (Amersham, RPN2106).

### RNA Isolation and Affymetrix GeneChip Hybridization, Normalization and Statistical Evaluation

RNA was purified with the RNeasy Lipid Tissue Mini kit (Qiagen, 74804). Quality control of total RNA was performed with an Agilent 2100 Bioanalyzer. RNA was quantified with a Nanodrop Spectrophotometer. cDNA synthesis was done with NuGEN’s Applause WT Amp Plus System (NuGEN, 5510–24) according to the manufacturer’s instructions, starting with 100 ng of total RNA. NuGEN’s Encore Biotin Module (NuGEN, 4200–12) was used to fragment 4 to 5 μg cDNA followed by Biotin-labeling according to the manufacturer’s instructions. For quality control of single-stranded cDNA (ST-cDNA) as well as fragmented cDNA Agilent 2100 Bioanalyzer and Nanodrop were used for exact quantification. Hybridization solution was prepared using the Affymetrix HWS Kit (Affymetrix, P/N 900720) according to NuGEN´ Applause WT Amp Plus System User Guide. The final concentration of cDNA was between 18 and 23 ng/μl. Hybridization was performed on Affymetrix GeneChips Exon 1.0 for 18+/-2 h at 45°C and 60 rpm in a rotating hybridization oven (Hybridization Oven 640, Affymetrix). Washing and staining was performed using the Affymetrix HWS Kit (Affymetrix, P/N 900720) with a fluidics station (GeneChip Fluidics Station 450, Affymetrix), which is controlled by Affymetrix´ software GeneChip Operating System (GCOS) v1.4. Scanning was performed with the GeneChip Scanner 3000 7G (Affymetrix), which is controlled by Affymetrix´ GeneChip Command Console Software (AGCC). The expression profiling data have been deposited at Gene Expression Omnibus, accession code: GSE66616.

### Epithelial Mesenchymal Transition (EMT) Score Gene Signature in Cell Lines

Gene expression data for 1032 unique cell lines were obtained from CCLE [21] and purchased from Eurofin Panlabs. The CEL files were Robust Multi-array Average (RMA) normalized using the Affy Package in R 2.14/Bioconductor. Expression quality was assessed using the R/Bioconductor packages affy and affyPLM [22,23,24]. We used alternative CDF files provided by Brainarray (Version 16.1.0, HGU133Plus2_Hs_ENTREZG), representing expression levels in a one-probe set one-gene fashion for 18927 Entrez Gene identifiers [25]. We summarized normalized log2 expression levels of cell line replicates as means and performed batch effect correction of the cell line expression data using Combat [26]. Subsequently, we performed gene-wise scaling of expression values by subtracting the mean and dividing by standard deviation over the whole cell line panel. Finally, we obtained the average of the genes in the EMT-signature [27] which is based on a meta-analysis of cells transfected with EMT inducing genes (GSG, SNAI1, TGFB1, TWIST1) [28].

### Ingenuity Pathway Analysis

RNA levels of mixed co-culture lysates (artificial mix of two mono-cultures) were compared to RNA levels derived from a real co-culture to calculate the fold changes of differentially regulated genes in a co-culture of tumor cells with different FBs. A list of upregulated genes was prepared for each co-culture pair (NCI-H1437+HDF, NCI-H1437+WI-38, NCI-H1437+NF1, NCI-H1437+CAF1, Calu-1+HDF, Calu-1+WI-38, Calu-1+NF1 and Calu-1+CAF1; FC>1.5 and p<0.01). Another set of overlapping upregulated genes in all NCI-H1437 co-cultures and a set of overlapping upregulated genes in all Calu-1 co-cultures was created and further analyzed with the Ingenuity Pathway Analysis software. The top ranked Ingenuity networks including the most deregulated molecules from the differential expression analysis were exported for further analysis. The Ingenuity Canonical Pathway was performed in parallel, which displays the most significant canonical pathways across the entire dataset, and across multiple datasets when reviewing comparison analysis. The significance value for the canonical pathways is calculated by Fisher's exact test right-tailed. The significance indicates the probability of association of molecules by random chance alone.

### Three Dimensional Cell Culture

The protocol used was adapted from Dolznig *et al*. [29]. Briefly, cells were seeded in 96-well Ultra-low attachment U-shaped plates (SIGMA Aldrich, LS7007-24EA) and incubated for 72 h at 37°C. The cell aggregates formed spheroids, were collected and embedded into rat tail collagen I (BD, 354236), which had been diluted according to the manufacturer’s protocol, except that distilled water was replaced by a 1.6% Methylcellulose (VWR, K390-250G) solution.

### Measurement of Single Cell Invasion and Collective Invasion Branches

Numbers of invasive structures were determined using ImageJ64 software. Coordinates of spheroid center, every invasive tip and single cell were exported to Microsoft Excel. Numbers of collective invasion branches (CIB) and single cells (single cell invasion; SCI) were counted and illustrated in bar charts. The phenotype of single cell and collective invasion was described by Friedl *et al*. [30].

### Statistical Analysis

All data shown represent mean ± standard error of the mean (SEM). Statistical analyses were performed using unpaired Student’s t-test. Significance levels are indicated as follows: *p<0.05, **p<0.01, ***p<0.001, ****p<0.0001; n.s.: not significant.

## Results and Discussion

### Selection of a non-invasive and invasive non-small cell lung cancer (NSCLC) cell line pair

**Fig 1:**
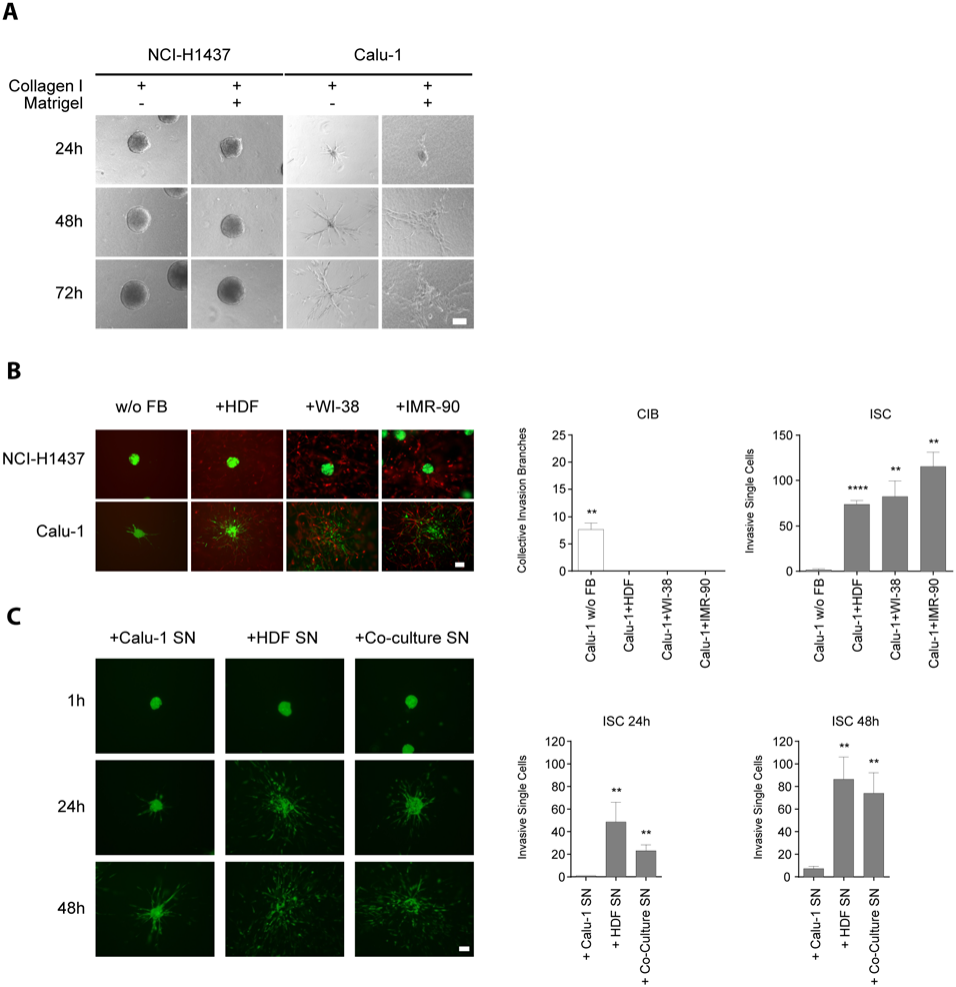
Lung cancer spheroid invasion into various matrices with and without stromal fibroblasts. (A) Light microscopy pictures were taken after one, two and three days of growth on either collagen I or collagen I/ matrigel matrices. Neither the non-invasive NCI-H1437 nor the invasive Calu-1 cell line changed their respective growth behavior in the presence of different matrices. Scale bar = 100 μm. Pictures are representative of at least three independent experiments. (B) GFP expressing NCI-H1437 and Calu-1 spheroids (green) were embedded into collagen I as mono-cultures (w/o FB) or grown as co-cultures with RFP (red) expressing human dermal FBs (HDFs), WI-38 (human embryonic lung FBs) or IMR-90 (human lung neonatal FBs). Pictures were taken after 24 h of incubation. Numbers of Calu-1 collective invasion branches (CIB, white bars) and the numbers of Calu-1 invasive single cells (ISC, grey bars) are depicted on the right (n = 3). Statistical analysis was performed on the means of ISC/CIB by unpaired comparison with Calu-1 w/o c using Student’s t-test (**p<0.01, ****p<0.0001). Experiments were repeated at least three times. (C) Fluorescent microscopy (Ex: 482 nm/Em: 502 nm) of 3D Calu-1 spheroid cultures (GFP expressing) incubated with supernatants (SN) derived from a Calu-1 mono-culture (+ Calu-1 SN), from an HDF mono-culture (+ HDF SN) or from a Calu-1/HDF co-culture (+ Co-culture SN) after 24 h and 48 h. Numbers of ISC are depicted on the right (n = 3). Unpaired comparisons of Calu-1 SN mean values with HDF SN and Co-culture SN were performed using Student’s t-test (**p<0.01). Scale bar = 100 μm.

In order to select an appropriate lung cancer cell line pair exhibiting the most polarized growth phenotypes (invasive *vs*. non-invasive), we first analyzed 10 different NSCLC cell lines for 3D spheroid formation (S1 Fig). All tumor cell lines constitutively expressed GFP for live cell imaging. After embedding the 3D spheroids into a collagen I matrix, invasion was observed in some of the 3D spheroid models after 24 h of incubation. Invasion was most prominent in Calu-1 cells whereas other cell lines such as NCI-H1437 exhibited non-invasive growth characteristics. As it is known that laminin-rich ECM can influence the invasive growth behavior [31,32] we analyzed the robustness of this growth phenoptype in laminin-containing (collagen I + matrigel) *vs*. in laminin lacking (collagen I alone) matrices. Importantly, both conditions retained the collective invasion phenotype of Calu-1 (see Fig 1A) and similarly non-invasive cells remained non-invasive independent of the matrix composition (NCI-H1437). Based on their phenotypically robust growth characteristics these two cell lines were selected for further studies. To set up organotypic 2D and 3D co-culture models for these two cell lines, Calu-1 and NCI-H1437 were co-cultured with various types of fibroblasts (FBs). FBs stably expressing RFP were used to allow simultaneous monitoring in co-cultures with GFP-expressing tumor cells. Interestingly, when Calu-1 cells were co-cultured with different FBs, Calu-1 cells dramatically changed their invasive phenotype from collective invasion to a more scattered phenotype (single cell invasion). The cellular characteristics of single cell and collective invasion was described by Friedl *et al*. [30] and served as a basis for measurement of single cell invasion (SCI) and collective invasion branches (CIB). In contrast, the growth behavior of the non-invasive cell line NCI-H1437 remained unaffected by FBs when co-cultured (Fig 1B). Co-cultivation of Calu-1 with normal lung derived WI-38 and IMR-90 led to a slightly more pronounced single cell invasion phenotype as compared to HDFs. In addition, primary lung derived NFs and CAFs also led to a highly scattered phenotype of Calu-1 spheroids (S2 Fig). In a reductionist approach HDFs were chosen for establishing a modular 2D cellular system with NCI-H1437 or Calu-1 representing the tumor and HDFs the stroma. Interestingly, supernatants derived from HDF mono-cultures and Calu-1/HDF co-cultures were sufficient to trigger single cell invasion of Calu-1 in 3D (Fig 1C), whereas control Calu-1 conditioned medium did not. Similar effects were shown with supernatants derived from 2D co-cultures with HDFs indicating that soluble paracrine factors were responsible for this effect.

### The tumor-stroma interaction models recapitulate MET activation and HDF-derived HGF is responsible for the single cell invasive phenotype of Calu-1 in co-cultures

**Fig 2:**
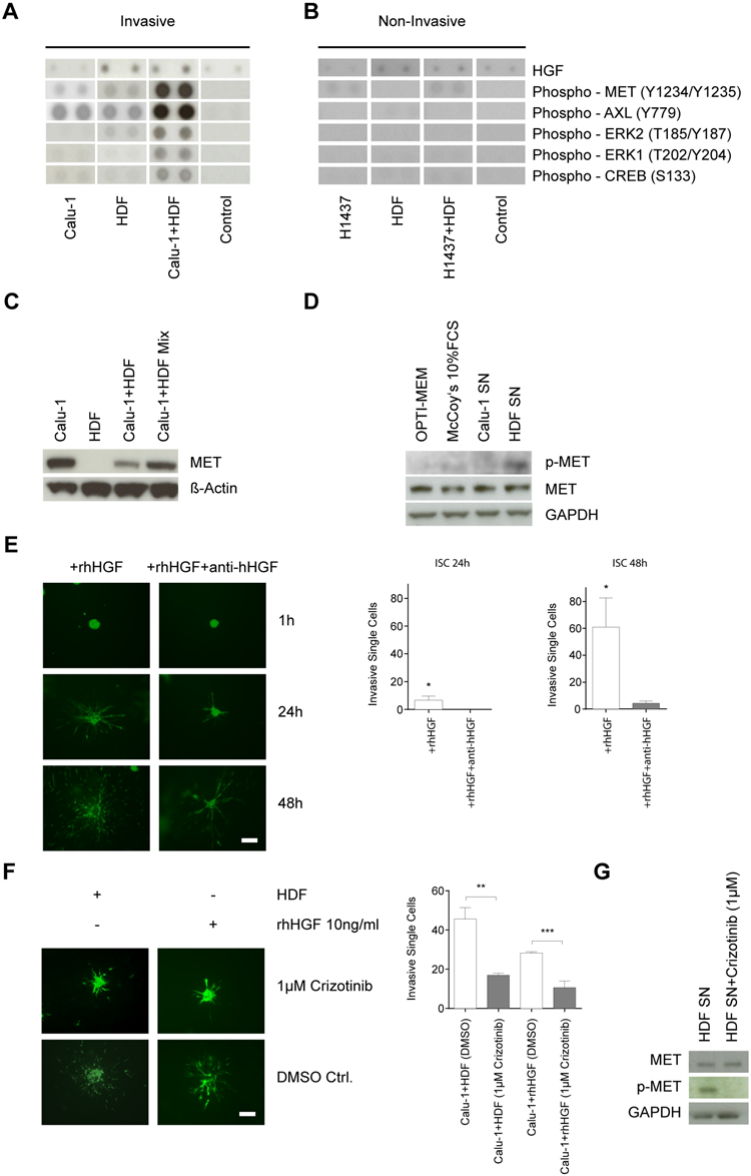
The HGF-cMET axis is recapitulated in the Calu-1 FB co-culture model and induces single cell invasion. (A) and (B) Proteome analysis of cell lysates and supernatants derived either from mono-cultures (Calu-1, NCI-H1437 (H1437), HDF) or from a 2D co-culture (Calu-1+HDF, H1437+HDF). All samples were taken from 24 h cultures as triplicates. For detection of ERK1(T202/Y204), ERK2(T185/Y187) and CREB(S133) the Phospho-MAPK Array (R&D) was used, for AXL(Y779) and MET(Y1234/Y1235) the Phospho-Receptor Tyrosine Kinase Array (R&D), lysis buffer served as control. For HGF the RayBio Cytokine Antibody Array was used, McCoy’s medium plus 10% FCS served as control. (C) MET Western blot analyses utilizing whole cell lysates from mono-cultures (Calu-1, HDF) and from corresponding 2D co-cultures (Calu-1+HDF). Calu-1 and HDF mono-culture lysates were artificially mixed (Calu-1+HDF Mix) to serve as control. (D) p-MET Western blot analyses of whole cell lysates from Calu-1 mono-cultures prior starved in OPTI-MEM for 6 h. Cells were harvested after 15 min of incubation in supernatants (SN) from Calu-1 or HDFs. OPTI-MEM and McCoy‘s 10% FCS medium served as a control. (E) Addition of either recombinant human HGF (rhHGF; 10 ng/ml; left panel) or rhHGF plus anti-hHGF antibody (1 μg/ml; right panel) in a 3D assay (n = 3). The number of invasive single cells (ISC) is depicted on the right for 24 h and 48 h of incubation (n = 3) (Student’s t-test: *p<0.05) (F) Calu-1 spheroids cultured with HDFs (left panel) or with 10 ng/ml recombinant human HGF (rhHGF, right panel). The upper panel shows treatment with 1 μM MET inhibitor crizotinib, whereas the lower panel serves as control (n = 3). Number of ISC is shown on the right. Statistical analysis was performed on the means of ISC by unpaired comparison with Calu-1+HDF (DMSO) or Calu-1+rhHGF (DMSO) samples using Student’s t-test (**p<0.01, ***p<0.001). Scale bar = 100 μM. (G) Calu-1 cells were starved in OPTI-MEM for 6 h and then incubated with HDF conditioned medium (HDF SN) alone or together with 1 μM of crizotinib (HDF SN+crizotinib 1μM). Cells were harvested after 15 min of incubation. Glyceraldehyde 3-phosphate dehydrogenase (GAPDH) was used as a loading control. p-MET = Phospho-MET (Y1234/Y1235).

As activation of the HGF/MET signaling pathway in NSCLC cell lines was previously shown *in vitro* [33] we wanted to confirm this in our co-cultures to proof the validity of our *in vitro* model. Therefore, we analyzed cell lysates from 2D-derived mono- and co-cultures on Phospho-MAPK and Phospho-Receptor Tyrosine Kinase (RTK) Arrays. In addition, the corresponding supernatants were subjected to Cytokine Array profiling. Indeed, in our co-culture model activation of MET was recapitulated (Fig 2A). In parallel, activation of AXL and downstream activation of ERK1/2 and CREB was observed. However, such an activation status was not observed in the corresponding NCI-H1437 co-culture (Fig 2B). Since other receptor tyrosine kinases on the array were not found to be activated such as VEGFR3, VEGFR1, c-Ret, EGFR, IGF-IR etc. it is very likely that mainly the HGF-MET-AXL-ERK1/2-CREB-axis contributes to the single cell invasive phenotype of the NSCLC cell line Calu-1.

Activation of MET upon co-cultivation did not allow for discrimination of whether HGF-triggered signaling is turned on in the FBs or in the cancer cells (Fig 2A). Therefore, we prepared whole cell lysates from both Calu-1 and HDF mono-cultures and from the corresponding co-cultures followed by Western blot analysis for assessment of total MET content. MET expression was found only in the Calu-1 cultures and not in the HDFs (Fig 2C). In addition, starved Calu-1 cells stimulated with HDF-conditioned media exhibited an activating phosphorylation of MET (Fig 2D) which is not observed in the non-invasive cell line NCI-H1437 (S3A Fig). One explanation for this finding is the lower expression level of MET in NCI-H1437 compared to Calu-1 (S3B Fig). Furthermore, by applying human recombinant HGF to Calu-1 mono-cultures a similar single cell invasion phenotype could be induced (Fig 2E, left column) as shown for the Calu-1/HDF co-culture (Fig 1B) or the incubation with FB conditioned medium (Fig 1C). Intriguingly, addition of a neutralizing HGF antibody (Fig 2E, right column) or treatment with the MET inhibitor crizotinib (1 μM) (Fig 2F and 2G) reversed the single cell invasion phenotype to the collective invasion type. In contrast, co-culturing two additional invasive cell lines (NCI-H157, NCI-H226) used for cytokine profiling (see further down) did not lead to a change in their invasive phenotype (S4 Fig).

The HGF/MET axis has been shown to play an important role in dissemination and metastasis of several different tumor types such as hepatocellular [34] and breast carcinomas [35]. In the context of NSCLC, MET has been mainly shown to contribute to escape from EGFR-inhibitor treatment and ALK-inhibitor based therapies in ALK-fusion containing tumors [36,37]. Recently, it was demonstrated that gemcitabine inhibits micrometastasis of NSCLC by targeting the EpCAM-positive circulating tumor cells via the HGF/MET pathway [38]. The direct involvement of the HGF/MET axis in the invasiveness of NSCLC cell lines was previously shown *in vitro* [33,39]. However, we demonstrate for the first time that HGF/MET-induced single cell invasion of lung tumor cells can be correlated with their invasive status and with the change in their invasive growth behavior in an *in vitro* 3D co-culture model (e.g. collective invasion *vs*. single cell invasion). As the HGF/MET-induced single cell invasion was only observed in one out of three invasive NSCLC cell lines (Fig 1 and S4 Fig), the involvement of this molecular axis might only contribute to invasiveness in a subset of lung tumors or during a distinct stage of tumorigenesis. In addition to our study and that from Nakamura [33], where FBs are an important source of HGF, Wang and colleagues identified tumor-associated macrophages (TAMs) as the main source for HGF [40]. Therefore, both cell types may be important producers of HGF presumably depending on the tumor type and stromal composition.

### Differential expression analysis reveals upregulation of genes mainly involved in tissue remodeling and inflammation (NFκB-related) in co-cultures of the invasive cell line Calu-1

**Fig 3:**
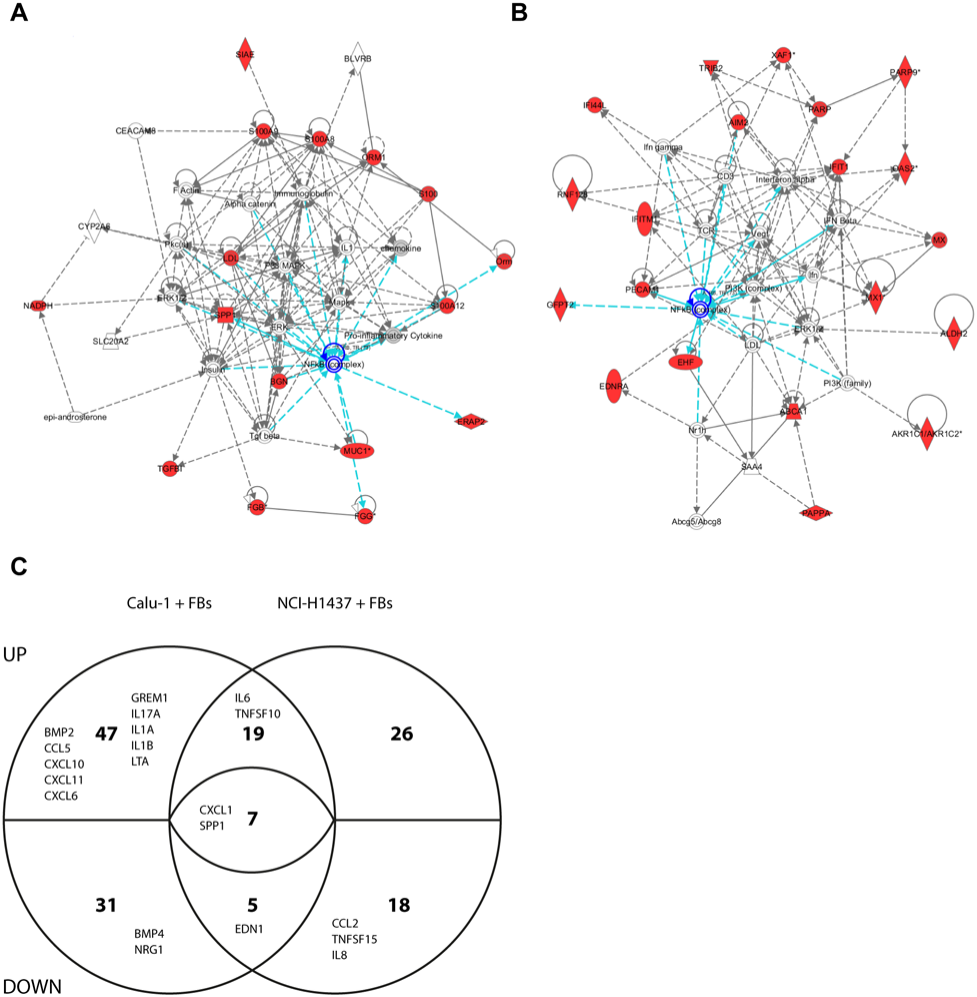
Transcription network analysis (Ingenuity) with genes specifically upregulated in co-cultures. (A) Transcription network based on genes upregulated in all four co-cultures of the non-invasive lung cancer cell line NCI-H1437. (B) Same as in A, but for co-cultures of the invasive lung cancer cell line Calu-1. Genes marked in red were found to be upregulated in co-cultures. FBs used: HDFs, WI-38, NF1 or CAF1, respectively. Selection parameters were: FC>1.5 and p<0.01. (C) Venn-diagram of all differentially regulated NFκB-linked genes (indicated by numbers) found at least once in a co-culture of the invasive Calu-1 or the non-invasive NCI-H1437 cell line with HDF, WI-38, NF1 and CAF1 (FC<-1.5 or FC>1.5 and p<0.01). Gene symbols indicate those genes exhibiting a cytokine activity (AmiGO2 gene ontology platform genes; Term: cytokine activity, Ontology source: molecular_function, Accession: GO:0005125, User filters: taxon_closure_label: Homo sapiens). Out of the seven genes shown in the center of the diagram two (OLR1 and SPP1) are upregulated and five (CASP1, CXCL1, LEF1, PLAU and TFPI2) are downregulated in NCI-H1437 co-cultures and *vice versa* in Calu-1 co-cultures.

Having established co-culture models and identified the activation of MET signaling as a major contributor to the induction of the single cell invasion phenotype in Calu-1, we next performed a global analysis of the molecular mechanisms involved in tumor-stroma crosstalk using expression profiling. Accordingly, we compared mRNA expression profiles of the non-invasive tumor cell line (NCI-H1437) with the invasive (Calu-1) both co-cultured with different FBs such as HDFs, WI-38 (fetal human lung), patient derived NFs (normal FBs) and CAFs (cancer-associated FBs). For a detailed description of all cell lines see S1 Table. In order to identify mRNAs that are specifically induced upon co-culturing, we followed the experimental setup as depicted in S5 Fig. Lysates from tumor cell mono-cultures were mixed with lysates from FB mono-cultures and designated as mono-culture mix. This artificial “mix” of RNA lysates allowed us to exclude additive effects of RNA levels. The same number of tumor cells and FBs used in the mono-cultures were used for generating the co-culture samples. All experiments were performed in biological triplicates. The collected RNA samples were further processed for whole genome Affymetrix GeneChip analysis. Based on this modular culture system [29] we were able to distinguish between mRNA expression levels in mono-cultured cells and mRNA levels specifically changed in co-cultures. RNA levels of mixed co-culture lysates were compared to RNA levels of real co-culture lysates to calculate the fold changes of differentially regulated genes within a co-culture of tumor cells and different FBs. First, upregulated genes for each of the two co-cultures with different FBs were identified. Secondly, a list of overlapping upregulated genes present in all co-cultures of NCI-H1437 or Calu-1 was generated and further analyzed with the Ingenuity Pathway Analysis software. In both co-cultures the top-ranked network identifies NFκB as an important hub (Fig 3A and 3B; marked in blue). In addition, co-cultures with Calu-1 exhibit interferon as a second important hub (Fig 3B). In this context it is of interest that IFNα/β stimulates NFκB DNA binding and NFκB-dependent transcription promoting cell survival in lymphoblastoid cells [41]. Convergence of the NFκB and interferon signaling pathways was described in the context of antiviral defense [42]. Whether such a convergence also holds true for the co-culture dependent signature remains to be determined in future experiments. As the NFκB hub was identified in both co-cultures (FBs plus invasive or non-invasive tumor cell line) we decided to focus on the NFκB-driven signature.

A considerable proportion (~8–14%) of all deregulated genes belong to the group of genes either involved in the regulation of NFκB or are themselves target genes of NFκB (Table 1; genes which are associated with NFκB are listed in S2 Table). Although both invasive and non-invasive tumor cell lines trigger the expression of genes linked with NFκB in their co-cultures, only the co-cultures of the invasive Calu-1 line led to the induction of a variety of cytokines (Fig 3C). In parallel, an Ingenuity Canonical Pathway analysis was performed displaying the most significantly deregulated canonical pathways across multiple datasets (Table 2). Comparison of upregulated genes from the top ranked canonical pathways in all four different co-cultures (Table 2) again revealed significantly more changes in co-cultures of the invasive cell line Calu-1. This indicated that the level of invasiveness of a tumor cell line determines the induction profile of genes rather than the type of FBs used. Intriguingly, again half of these genes are linked to NFκB (S3 Table). The majority of upregulated genes in the NCI-H1437 co-cultures are genes of the extracellular matrix such as various collagens, fibronectin, complement components and orosomucoid 2 (ORM2).

**Table 1:** Percentage of NFκB-related target genes within all differentially regulated genes in a co-culture of Calu-1 or NCI-H1437 with HDF, WI-38, NF1 or CAF1.

	Calu-1+HDF	Calu-1+WI-38	Calu-1+NF1	Calu-1+CAF1	H1437+HDF	H1437+WI-38	H1437+NF1	H1437+CAF1
NFκB related genes/ All differentially regulated genes	22/181	48/499	52/426	79/1029	39/400	21/259	48/351	23/254
Percentage	(12.15%)	(9.62%)	(12.21%)	(7.68%)	(9.75%)	(8.11%)	(13.68%)	(9.06%)

Selection parameters were: FC≥1.5 or FC<-1.5 and p≤0.01.

**Table 2:** Ingenuity Canonical Pathway Analysis.

Category	Pathway	Cell line+CAF	Cell line+HDF	Cell line+NF	Cell line+WI-38
**NCI-H1437 Canonical Pathway Analysis**	Acute Phase Response Signaling	6,01	1,65	8,79	3,42
	Intrinsic Prothrombin Activation Pathway	5,09	6,04	5,11	3,82
	Atherosclerosis Signaling	2,64	3,92	4,50	
	Coagulation System	3,29	2,88		3,57
	Extrinsic Prothrombin Activation Pathway	4,25	2,31		4,53
	LXR/RXR Activation	3,59		3,66	4,04
	Role of IL-17A in Psoriasis	2,83	4,19		3,01
**Calu-1 Canonical Pathway Analysis**	Granulocyte Adhesion and Diapedesis	10,40	1,50	8,26	4,28
	Hepatic Fibrosis / Hepatic Stellate Cell Activation	5,42	3,65	6,53	2,62
	Inhibition of Matrix Metalloproteases	3,72	1,98	4,25	2,19
	Interferon Signaling	7,79	3,49	5,84	7,50
	Agranulocyte Adhesion and Diapedesis	7,33		7,92	3,33
	Communication between Innate and Adaptive Immune Cells	4,42		3,37	2,71
	Leukocyte Extravasation Signaling	5,39		3,55	2,48
	Role of Macrophages, Fibroblasts and Endothelial Cells in Rheumatoid Arthritis	3,46		3,35	3,69
	Role of Osteoblasts, Osteoclasts and Chondrocytes in Rheumatoid Arthritis		1,25	4,60	2,23
	Role of Pattern Recognition Receptors in Recognition of Bacteria and Viruses	5,13		5,06	6,40

The top ranked Ingenuity canonical pathways resulting from differentially upregulated gene expression analysis (FC≥1.5 and p≤0.005) of the respective co-cultures were compared with each other. Calculation of significance was done by Fisher's exact test right-tailed. Canonical pathways exhibiting significant changes in at least three out of the four co-cultures are depicted. Numbers represent—log(p-value).

Calu-1 co-cultures exhibit a gene profile linked with tissue remodeling such as CXCL10, 11 and MMP1, 14 or interleukins (IL1A,B; IL6), interleukin 1 receptor (IL1R1) or with interferon-induced proteins (IFIT1 and3, IFITM1, IRF9, MX1) as well as EDNRA, FPR1, VCAM1, VEGFA, PECAM1, TFPI2 and THBS2. In particular, EDNRA [43], FPR1 [44] and VCAM1 [45] have been shown to essentially contribute to the invasiveness of tumors. In general, co-cultures with the invasive cell line Calu-1, but not with NCI-H1437, led to expression of genes involved in tissue remodeling, inflammation and tumorigenesis.

### Identification of “cytokine fingerprints” in co-cultures

As transcription profiling had revealed that a significant portion of deregulated genes in co-cultures are cytokines being either direct or indirect targets of NFκB, we next performed a detailed cytokine profiling analysis at the protein level. Following the protocol as outlined in S5 Fig. cell supernatants were prepared and subjected to Human Cytokine Array analysis. Due to variability of the signal on the arrays the following screening strategy was applied: For each supernatant four biological replicas were performed and three different exposure times were used to achieve a dynamic range of the signals.

**Fig 4:**
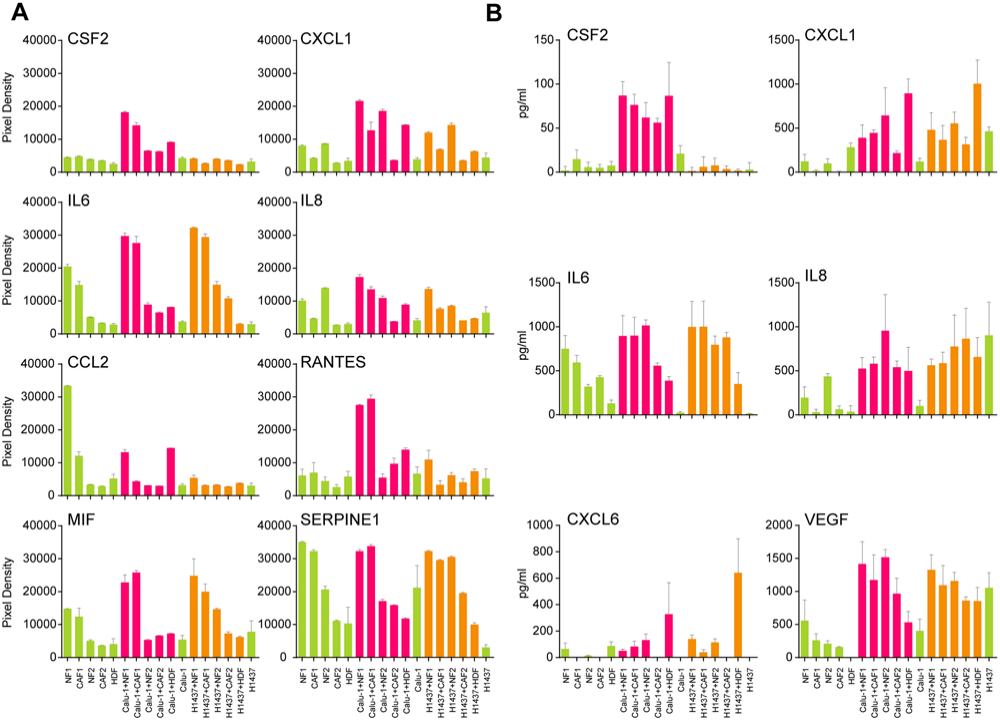
Cytokine Array- and ELISA-based “cytokine fingerprint” of supernatants derived from different mono- and co-cultures. Calu-1 and NCI-H1437 tumor cells were co-cultured with HDFs and two patient-derived NF/CAF pairs (NF1/CAF1, NF2/CAF2) for 24 h. Supernatants from mono- (green) and co-cultures (purple: Calu-1 and orange: NCI-H1437) were collected and tested for the indicated cytokines with at least three biological replicates, each representing two technical replicates (Cytokine Array), or three biological replicates, each representing three technical replicates (ELISA). (A) Cytokine Array: Relative pixel counts on the Y-axis. (B) ELISA: The cytokine level is depicted on the Y-axis [pg/ml]. Statistical analysis was performed by using Student’s t-test (for p-values see S4 Table).

Utilizing two different cytokine array platforms (RayBio and R&D) 17 (C5a, GCP2/CXCL6, G-CSF, CSF2/GM-CSF, GROa/CXCL1, IGFBP-1, IGFBP-2, IL1B, IL2, IL5, IL6, IL8, MCP-2, M-CSF, sICAM-1, uPAR and VEGF) out of 130 tested cytokines/growth factors were shown to be specifically induced upon co-culturing of HDFs either with the invasive tumor cell line Calu-1 or with the non-invasive NCI-H1437. Supernatants of co-cultures with FBs derived from two patient samples (NF/CAF1 and 2) (Fig 4A) showed similar patterns of induction. In order to generate a “cytokine fingerprint” of the different co-cultures we focused on the most upregulated cytokines and quantitatively analyzed those by ELISA. This showed that CSF2, CXCL1, IL6 and IL8 had the most pronounced induction profiles (Fig 4B). Notably, CXCL6 exhibited a strict dermal FB-specific induction profile: Regardless of which tumor cell line was used, only co-cultures with HDFs led to a strong induction of CXCL6 (Fig 4B). In contrast, CSF2/GM-CSF was only induced in co-cultures with the invasive cell line Calu-1 regardless of the partnered FB type whereas no induction of CSF2 was observed in all co-cultures with the non-invasive cell line NCI-H1437 (Fig 4A, 4B and S4 Table). In the case of IL6, the NF1/CAF1 pair already exhibited high expression whereas other FBs such as HDFs and the NF2/CAF2 pair show rather low expression levels. Therefore, the expression of IL6 in co-cultures with HDFs or with the NF2/CAF2 pair can be regarded as inducible. Perhaps this reflects the heterogeneity of the tumor-stroma crosstalk as shown in an *in vitro* murine lung cancer model [46]. In contrast, VEGF was already expressed in the non-invasive cell line NCI-H1437 but was induced in Calu-1 specifically upon co-culturing with all lung derived FBs. A similar profile was identified for IL8 (Fig 4B). In general, there seems no preference for a certain FB type for the induction of cytokines except for CXCL6.

So far very little was known about the specific cytokine secretome of lung tumor stroma. In an *in vitro* system, a peptide comprising the type III repeat of fibronectin (FnIII-1c) could induce the expression of CXCL1 and IL8 in adult human lung FBs [47]. In addition, a murine lung adenocarcinoma cell line harboring mutant KRAS co-cultured with various types of stromal cells was associated with secretion of CXCL1, among other proteins, involved in angiogenesis, inflammation, cell proliferation and EMT [46]. Interestingly, our co-cultures of both Calu-1 (KRAS^mut^) and NCI-H1437 (KRAS^wt^) led to induction of CXCL1 in HDF whereas Calu-1 triggered the induction of CXCL1 also in the organ specific FBs upon co-cultivation (Fig 4B). Recently, CXCL1 was identified as a potent stromal protein marker of dysplasia-carcinoma transition in sporadic colorectal cancer [48]. RANTES and MIF show a rather patient specific profile as strong induction is only seen with NF1 or CAF1 co-cultures and Calu-1. In addition, MIF is also induced upon co-cultivation with NCI-H1437 and NF1 or CAF1 (Fig 4A). For SERPINE1 no specific co-culture effect was observed (Fig 4A).

### CSF2 is only induced in co-cultures with tumor cells exhibiting an EMT signature

As CSF2 exhibits the most restrictive cytokine induction profile in Calu-1 co-cultures (Fig 4), we next addressed the question whether this was a general phenotype associated with the invasive capacity of lung cancer cells. In addition, we tested whether the induction of CSF2 might be linked to a certain gene signature. In order to determine this, we selected two additional pairs of NSCLC tumor cell lines based on both the presence and the absence of an EMT signature and the ratio of E- *vs*. N-cadherin. The EMT signature was first described by Taube *et al*. who identified a core EMT transition gene-expression signature which is associated with metaplastic breast cancer subtypes and negatively correlates with pathological complete response [28]. This EMT signature was also applied in glioblastoma multiformis (GBM) showing again a correlation with the mesenchymal and proneural subtype of GBM [49]. In addition, the EMT gene expression signature predicts resistance to EGFR and MEK-targeted therapies in cell lines and almost all solid tumor patient samples [27], as well as resistance to EGFR and PI3K inhibitors [50]. Finally, transcription profiling was used to identify genes involved in EMT utilizing a murine EpH4 model [51].

**Fig 5:**
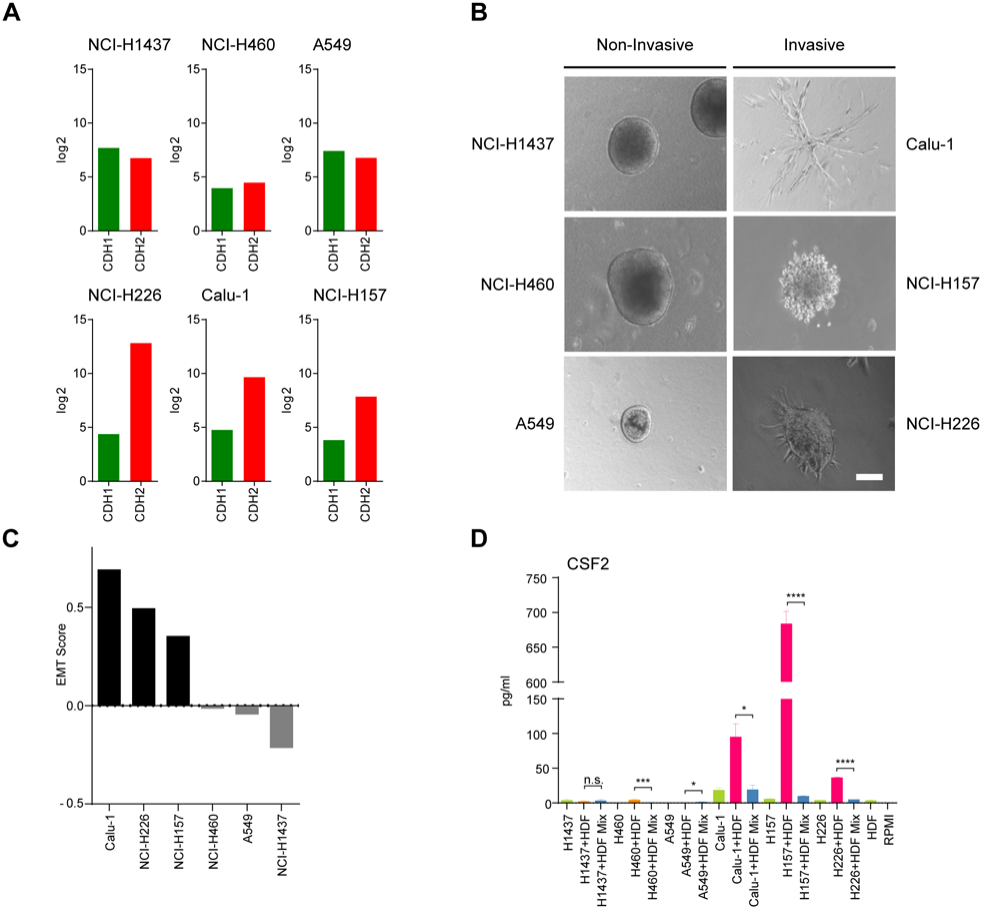
Growth behavior, ratio of E-cadherin *vs*. N-cadherin expression levels, and EMT score of three NSCLC cell line pairs and the CSF2 levels in supernatants of the respective mono- and co-cultures. (A) E- *vs*. N-cadherin log-2-transformed relative expression levels (Affymetrix HGU133 Plus 2). (B) Light microscopy pictures of three-dimensional spheroid cultures. Tumor spheroid aggregates were embedded into collagen I and pictures were taken after 72 h. NCI-H1437, NCI-H460 and A549 spheroids showed a non-invasive phenotype (left), whereas Calu-1, NCI-H157 and NCI-H226 spheroids revealed an invasive phenotype (right). Scale bar = 100 μm (n = 3). (C) EMT-score based on EMT signature gene set of Taube *et al*. [28] and Eddy *et al*. [27]. A set of EMT signature genes was used to rank invasive (black bars) and non-invasive (grey bars) cell lines according to their EMT score. (D) Levels of CSF2 [pg/ml] in supernatants of mono- (green) and co-cultures (purple: Calu-1 and orange: NCI-H1437). Experiment was performed in triplicates (n = 3). Invasive and non-invasive NSCLC cell lines have been co-cultured with HDFs. Mixes of supernatants of mono-cultures (blue) derived from the respective tumor cell lines and HDFs, respectively, served as controls. Statistical analysis was performed on the mean values by unpaired comparison using Student’s t-test (*p<0.05, ***p<0.001, ****p<0.0001; n.s.: not significant).

NCI-H1437, NCI-H460 and A549, which displayed a negative EMT score exhibited non-invasive growth in a collagen I matrix and showed an equal N-cadherin/E-cadherin ratio, whereas Calu-1, NCI-H157 and NCI-H226, which had high EMT scores and high N-cadherin/E-cadherin ratios followed the pattern of invasiveness (Fig 5A–5C). Upon co-culturing of all six tumor cell lines with HDFs only those co-cultures with the invasive tumor cell lines (Calu-1, NCI-H157 and NCI-H226) led to a massive increase in the expression of CSF2 as shown by ELISA of the respective supernatants (Fig 5D, right part). In contrast, none of the non-invasive tumor cells (NCI-H1437, A549 and NCI-H460) were able to trigger induction of CSF2 expression in co-cultures (Fig 5D, left part).

So far very little is known about the induction of CSF2/GM-CSF in lung cancer; Nonaka *et al*. demonstrated induction of CSF2 expression in nasal but not in pharyngeal, tracheal, bronchial and lung FBs [52]. In murine lung tumor models CSF2 stimulates growth and differentiation of myeloid progenitors, and generates potent, specific and long-lasting anti-tumor immunity [53]. On the other hand, CSF2 has been shown to increase production of matrix metalloelastase in tumor-infiltrating macrophages, which might result in a more aggressive cancer [54].

### Canonical NFκB signaling in co-cultured FBs is responsible for CSF2 expression

**Fig 6:**
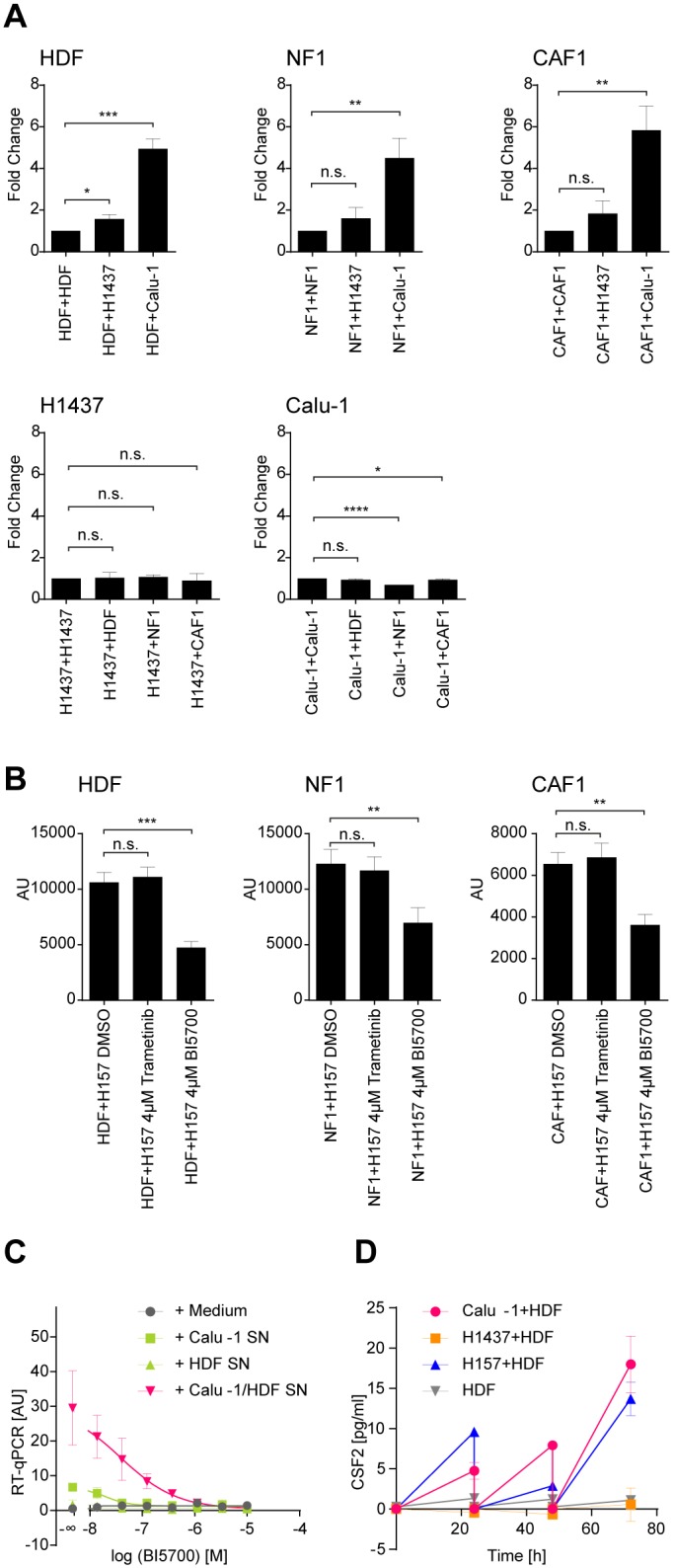
NFκB reporter activity and induction of CSF2. (A) Luciferase-based NFκB reporter activity in co-cultures of Calu-1 or NCI-H1437 with various FBs. 24 h after transfection of the respective cell type (depicted in the title of each graph) the NFκB-reporter plasmid-containing cell line was incubated with a non-transfected cell line as indicated on the X-axis for additional 24 h. The upper three graphs display studies with transfected FBs co-cultured with non-transfected tumor cell lines or with the non-transfected parental FB line, respectively. The lower two graphs exhibit the experiment with transfected tumor cell lines. Bars indicate fold changes of the Luciferase reporter signal mean values. For FBs lipofectamine transfection, for tumor cell lines electroporation transfection was applied. (B) NCI-H157 co-cultures were treated with trametinib and BI5700 for 24 h (4 μM each). The different FBs had been transfected prior with an NFκB-reporter plasmid. Luciferase units are depicted on the Y-axis. (C) BI5700 dose-dependent reduction of CSF2 transcription in HDFs. Relative CSF2 transcripts are depicted (RT-qPCR). Supernatant from a 24 h HDF mono- or Calu-1/HDF co-culture was transferred to an HDF mono-culture for another hour with or without BI5700 treatment. Incubation with RPMI 10% FCS medium served as a control. The level of CSF2 transcripts in the respective non BI5700-treated HDFs is indicated by [-∞]. Statistical analyses were performed on the mean values by unpaired comparisons using Student’s t-test (*p<0.05, **p<0.01, ***p<0.001, ****p<0.0001; n.s.: not significant). (D) ELISA for CSF2 (Y-axis [pg/ml]) of supernatants derived from mono- and co-cultures of invasive (Calu-1, NCI-H157) and non-invasive tumor cell lines (NCI-H1437) with HDFs. Supernatants were taken and replaced with fresh medium at the indicated time points (X-axis; time [h]). All experiments were performed in triplicates (n = 3).

So far, we have demonstrated that only invasive tumor cell lines co-cultured with FBs triggered the induction of CSF2 expression and that a high proportion of the deregulated genes in those co-cultures are closely linked with NFκB (Table 1 and S2 Table). Thus far, the experimental setup has not allowed us to distinguish between the various cell types in a co-culture. Hence we next assessed in which cell type NFκB signaling is turned on upon co-cultivation. For this we transiently transfected a luciferase-based NFκB-reporter plasmid into either the FBs or into the respective tumor cell line followed by co-cultivation with the corresponding non-transfected partner cell (e.g. NFκB-transfected Calu-1 cells were co-cultured with non-transfected HDFs and *vice versa*; Fig 6). Firstly, NFκB signaling was exclusively induced in FBs but not in tumor cells upon co-culture (Fig 6A). Secondly, NFκB signaling was significantly induced in the FBs co-cultivated with the invasive tumor cell line (Calu-1) but not with the non-invasive NCI-H1437 (Fig 6A). Finally, induction was not dependent on the type of FBs used (Fig 6A). Next we clarified whether the activation of the NFκB pathway in FBs was due to the canonical (via IKKß) or non-canonical signaling (e.g. via MEK) using selective inhibitors of IKKß (BI5700) and MEK1/2 (trametinib) [55,56,57]. FBs were transfected with the NFκB reporter plasmid prior to the treatment with specific inhibitors targeting either the canonical or non-canonical pathway. As seen in Fig 6B only the IKKß inhibitor BI5700 [55] was able to reduce the NFκB reporter signal in FBs. Conversely, trametinib, a potent and selective MEK inhibitor, did not produce this effect [56,57], suggesting that canonical signaling was responsible for NFκB activation in the FBs.

Consistently, incubation of HDFs with supernatants from Calu-1/HDF co-cultures caused a dramatic increase in CSF2 mRNA. However, no induction at all was observed in HDF-conditioned medium. Most importantly this expression could be reduced in a dose-dependent manner by addition of the IKKß inhibitor BI5700 (Fig 6C) with an estimated IC_50_ value of approximately 50 nM, strongly indicating that CSF2 was induced by canonical NFκB signaling in the FBs. Furthermore, co-cultures of Calu-1 or NCI-H157 with HDFs led to a long-term expression of CSF2 as demonstrated by replacing the supernatants of the respective cultures with fresh medium every 24 h (Fig 6D). In contrast, no induction of a constitutive expression could be detected in co-cultures with NCI-H1437 or in mono-cultures of HDFs (Fig 6D). Taken together, we provide clear evidence that NFκB signaling is induced exclusively in co-cultured FBs but not in partnered invasive tumor cells and that induction is not dependent on the type of FBs. Once induction occurs it remains constitutive as shown by the continuous secretion of CSF2 into the supernatant. This implies that the invasive tumor cell but not the non-invasive “steers” the induction of NFκB signaling. As suggested by the study with supernatants derived from co-cultures of invasive tumor cell lines (Fig 6C), the expression of CSF2 is achieved via a paracrine mechanism.

Both the canonical and non-canonical NFκB pathways were shown to play an important role in a variety of normal and pathological molecular mechanisms [58, 59, 60, 61,62]. shRNA-mediated inhibition of NFκB signaling in CAFs was previously shown to play an important role in murine tumor models [18]. In contrast to the common understanding that CAFs are the main drivers in the tumor-stromal crosstalk leading to increased malignancy [63,64] we showed that, in addition to CAFs, FBs of other sources (such as HDFs and NFs) can contribute to an inflammatory signature without being previously “educated” (see Fig 3). In summary, inhibition of the canonical NFκB pathway in the tumor stroma can be regarded as a feasible therapeutic approach. This holds particularly true for human tumors or tumor types exhibiting a strong inflammatory stromal compartment such as gastric, colon and ovarian cancer [53,65].

### Increased mRNA levels of cytokines (CSF2, IL6, IL8 and IL1B) and chemokines (CXCL1 and CXCL6) in co-cultured HDFs

**Fig 7:**
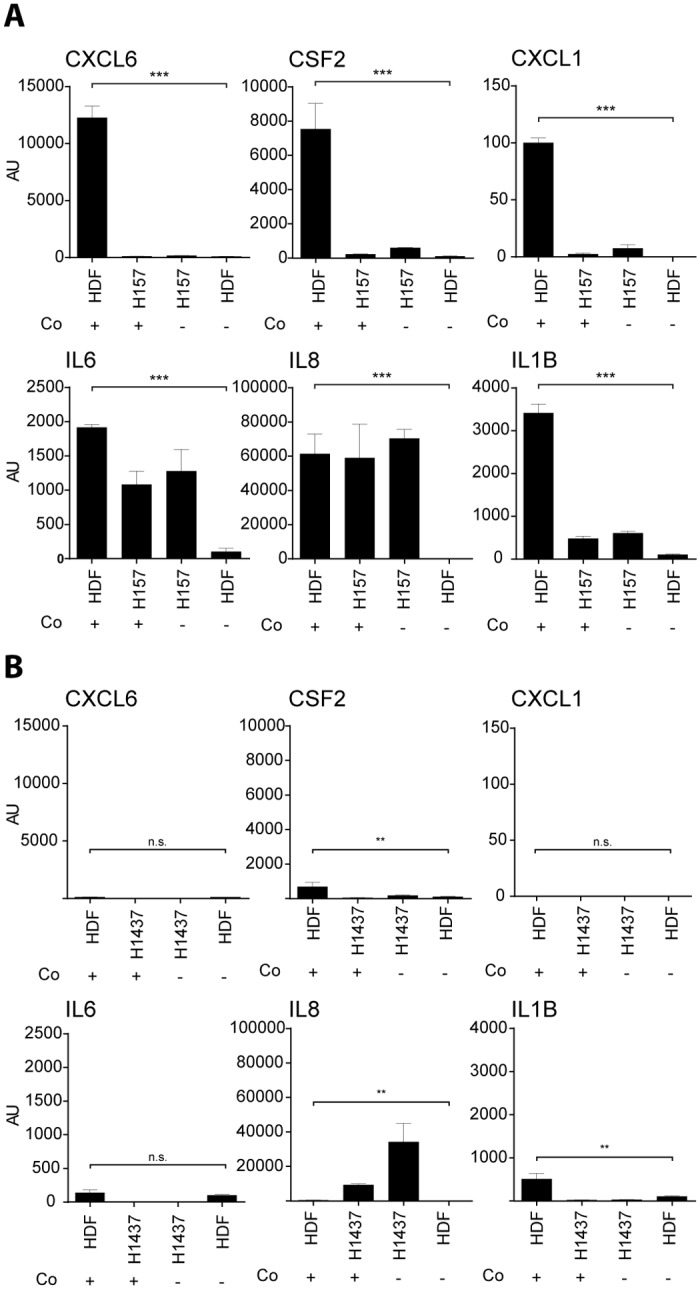
RT-qPCR for a set of cytokines (CSF2, IL6, IL8 and IL1B) and chemokines (CXCL1 and CXCL6) of total RNA samples derived from mono- and co-cultures in a transwell assay. The respective co- (+) or mono- (-) culture is indicated on the X-axis. The respective analyzed cytokine or chemokine is indicated in the header of each graph. Expression values are shown in arbitrary units (AU) and have been normalized to beta-2 microglobulin (B2M) mRNA copies. Experiments were performed in triplicates (n = 3). Statistical analysis was performed on the mean values by unpaired comparison of mono-cultured HDF and co-cultured HDF RNA samples by using Student’s t-test (**p<0.01, ***p<0.001; n.s.: not significant).

From the studies performed thus far it could not be deduced (except for CSF2) in which specific cell type the respective genes are induced upon co-cultivation and whether induction takes place at the transcriptional level. To address this question, we set up a transwell assay with the tumor cell lines NCI-H157 or NCI-H1437 cultured in the upper part and HDFs at the bottom of the transwell plate. A pore size of approx. 0.4 μm allowed an efficient exchange of proteins and soluble factors but prevented intermingling of the two cell types. Since the different cells were cultured in two compartments they easily could be separated at certain time points after co-culturing and analyzed independently from each other. mRNA prepared from separated cells of mono- and co-cultures were subjected to RT-qPCR. In line with our previous findings, co-cultures of NCI-H157 (invasive) and HDFs led to an increase of transcripts from CSF2, CXCL1 and CXCL6 exclusively in HDFs but not in the tumor cell line (Fig 7A upper panel). Similar results were obtained with patient-derived FBs co-cultivated with NCI-H157 (S6 Fig). In contrast and in agreement with our data co-cultures with the non-invasive NCI-H1437 did not exhibit such an induction profile (Fig 7B and S6 Fig). Likewise, IL1B, IL6 and IL8 got induced in HDFs but expression was also seen in the tumor cell line (Fig 7A lower panel). However, expression of IL1B, IL6 and IL8 in the tumor cell line was constitutive and could not be additionally induced upon co-cultivation with HDFs. Based on these findings it appears that the analyzed cytokines and chemokines are induced in HDFs at the mRNA level.

The reason why tumor cells constitutively express some cytokines such as IL6 and IL8 remains unclear. For instance, head and neck squamous cell carcinoma (HNSCC) has been tied to high cytokine expression both *in vitro* and in patients [66]. In particular, expression of IL6 in HNSCC is associated with increased invasiveness, as well as poorer patient prognosis and higher recurrence rates [67]. IL8 has been reported to play an important role in tumor progression and metastasis in a variety of human cancers, including lung cancers [68]. The presence of IL8 in tumors and the tumor microenvironment may contribute to tumor progression by regulating angiogenesis, cancer cell growth and survival, tumor cell migration, leukocyte infiltration and modification of immune responses. However, a detailed understanding of the contribution of a constitutive and/or the inducible expression of both IL6 and IL8 in tumor cells is still missing [69]. Perhaps studies with more complex co-cultures supplemented with additional cell types e.g. inflammatory cells or their precursors might lead to a deeper understanding of these molecular mechanisms. An example in this direction is shown further down.

### Discovery of signaling pathway response to co-culturing (multi-reporter assay)

In our co-cultures, induction of NFκB signaling was shown to be triggered in FBs but not in tumor cells upon co-cultivation (Fig 6). In order to determine which other signaling pathways are activated in co-cultures, we made use of the “Cignal Finder Reporter Array”. In brief, each array includes 45 “Cignal Reporter Assays” based on a dual-luciferase technology and respective controls in a 96-well plate format (for details see Materials and Methods). In the present study, we used reporter gene transfected FBs and either co-cultured them with non-transfected FBs (constitutive/basic reporter signal) or with non-transfected tumor cells (specific induction signal). A similar setup was used to identify signaling pathways induced specifically in the cancer cells upon co-culturing with FBs using reporter gene transfected tumor cells and non-transfected FBs.

**Fig 8:**
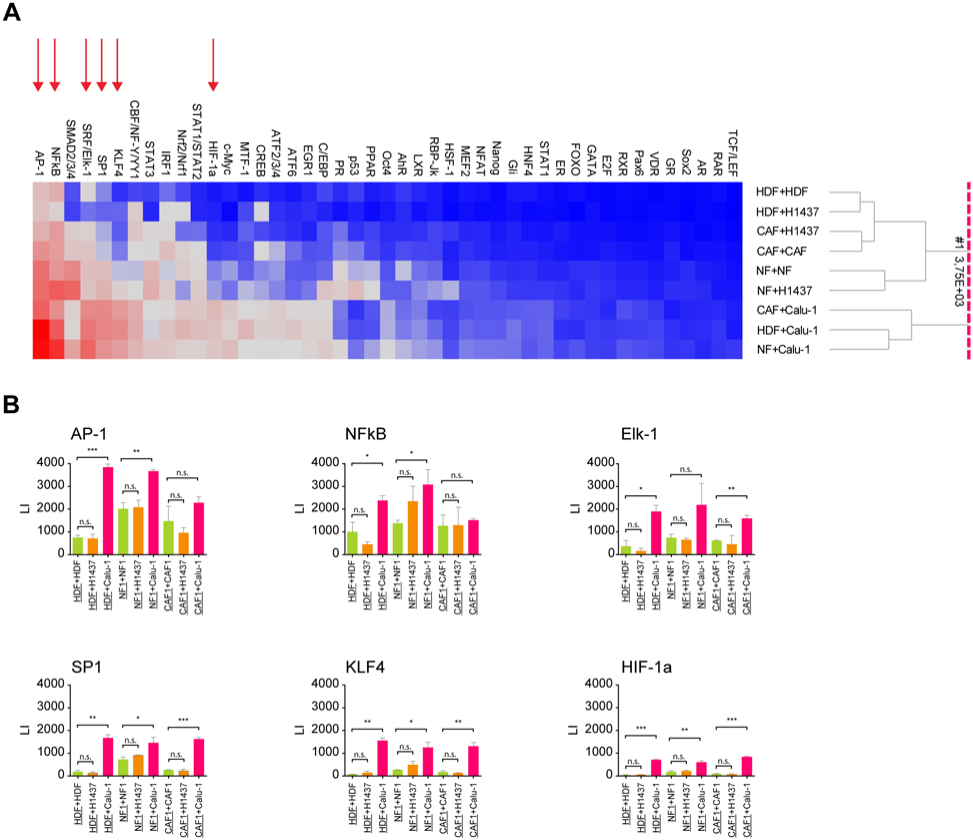
Multi-gene reporter array assay in co-cultures of reporter-construct-transfected FBs. The different FBs were either co-cultured with themselves or with NCI-H1437 or Calu-1 tumor cells. For each co-culture three independent experiments were performed. (A) Overview (heat map) on Luciferase signals of each of the 45 reporter constructs in transfected FBs (HDF, NF1 and CAF1). Reporters are indicated at the top. Culture conditions are depicted at the right side. Clustering method: UPGMA; distance measure: euclidean; ordering weight: average value. Red arrows indicate the six most significantly upregulated signaling pathways upon co-cultivation which are shown in detail in B. (B) Underlined FBs on the X-axis indicate the cell line which had been transfected with the corresponding reporter construct depicted in the header of each diagram. In all cultures either the same number of transfected FBs were co-cultured with the same number of non-transfected FBs (HDF+HDF, NF1+NF1 and CAF1+CAF1) or accordingly, with the same number of cells of non-transfected tumor cell line (HDF+Calu-1, HDF+H1437 etc.). Controls are depicted in green, Calu-1 co-cultures in purple and NCI-H1437 co-cultures in orange. Statistical analysis was performed by unpaired comparison of control samples (HDF+HDF, NF1+NF1, CAF1+CAF1) with respective co-culture samples by using Student’s t-test (*p<0.05, **p<0.01, ***p<0.001; n.s.: not significant). LI = luminous intensity.

The experiments confirmed our previous finding of induction of NFκB signaling in FBs upon co-cultivation. In addition to NFκB-signaling, multiple other signaling pathways were found to be induced in FBs including the AP-1, HIF-1a, KLF4, SP-1 and ELK-1 pathways (Fig 8). Once again, this is in agreement with our previous results (Figs 6 and 7) showing that only co-cultivation with the invasive tumor cell line Calu-1 led to a significant induction of reporter signaling in FBs. Interestingly, there was no significant difference in the level of inducibility of these six signaling pathways with regard to the type of FBs (CAFs, NFs or HDFs) used (Fig 8). In contrast, in both tumor cell lines, no change in the level of the reporter gene activity in any of the tested signaling pathways was detectable upon co-cultivation, however, there were a few signal transduction pathways already constitutively turned on in the tumor cells.

In order to get a deeper molecular understanding of the signals turned on in FBs, further experiments are clearly needed. AP-1, for instance, regulates gene expression in response to a variety of stimuli, including cytokines, growth factors and stress. In epithelial cells, the co-stimulation of AP-1 and NFκB has been shown to contribute to the induction of CSF2 [70]. A similar mechanism might operate in FBs.

KLF4, a member of the erythroid Kruppel-like factor (EKLF) multigene family plays a critical role in the differentiation of epithelial cells [71]. Depending on the target gene, KLF4 can function as both a repressor and activator of transcription [72]. To further muddy the waters, KLF4 can function as a tumor suppressor or as an oncogene depending on the tumor type. The paradoxical nature of KLF4 extends from its function to its intracellular levels. KLF4 has been reported to be upregulated in human HNSCC and downregulated in colorectal carcinoma [73,74]. Moreover, it has been implicated in the generation of induced pluripotent stem cells [75]. How these features of KLF4 may contribute to the crosstalk between tumor cells and FBs remains to be determined.

The same holds true for the ubiquitously expressed transcription factor SP1 which interacts with many other transcription factors such as MYC, EGR1 and STAT1 [76]. Of particular interest in the context of our finding is the observation that SP1 overexpression has been linked with fibrosarcoma and that downregulation of overexpressed SP1 protein in human fibrosarcoma cell lines inhibits tumor formation in murine models [77]. The transcription factor ELK-1 is a component of the ternary complex that mediates gene activity in response to serum and growth factors [78]. Recently, an increase in ELK-1 mediated signaling was found in breast myofibroblast-like CAFs. Most interestingly, those myofibroblast-like CAFs also exhibit elevated activation of NFκB and AP-1 [79].

The contribution of HIF-1α was recently shown to play an important role in breast cancer CAF/tumor cell crosstalk. Regulation of autophagy, glycolysis and senescence in CAFs via activation of HIF-1α and NFκB promotes tumor growth and survival [80,81]. Furthermore, HIF-1α functions as a tumor promoter in CAFs and as a tumor suppressor in breast cancer cells [82]. Whether HIF-1α has a similar function in lung cancer remains to be addressed.

### Co-culture of invasive tumor cells (NCI-H157) with HDFs recruits THP-1 macrophage like cells

Besides endothelial cells and FBs, the tumor microenvironment also harbors innate and adaptive immune cells [83]. It is generally accepted that in contrast to the original function of these immune cells, namely recognizing and attacking transformed cells, they contribute to tumor progression by promoting inflammation in an NFκB-dependent manner [18]. Chronic inflammation triggers cellular events that can promote malignant transformation of cells and carcinogenesis in a variety of tumor types such as colorectal, breast, prostate and cholangio carcinoma [84]. Nevertheless, not only how tumors promote inflammation but also how they engage inflammatory cells, is still unknown and intensively studied. In order to begin to address the question of chemoattraction of monocytes in co-cultures we made use of THP-1, a human monocytic leukemia cell line with characteristics of a pre-mature macrophage cell. The THP-1 cell line provides not only a valuable model for studying the mechanisms involved in macrophage differentiation, but also for exploring the influence of secreted cytokines on migration [85].

**Fig 9:**
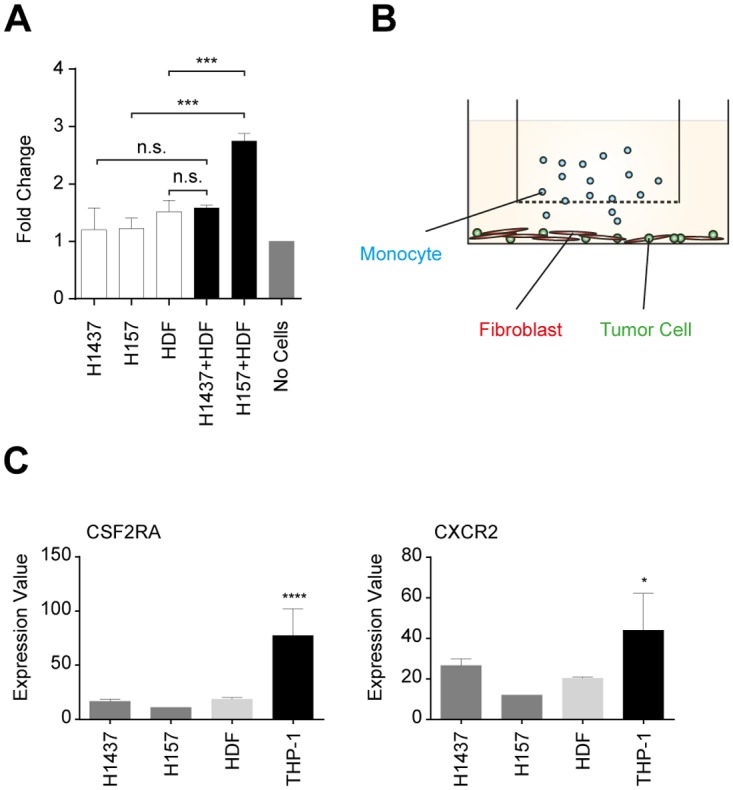
Transwell migration assay in a triple culture of a non-invasive (NCI-H1437) or an invasive tumor cell line (NCI-H157), HDFs and THP-1, and relative expression levels of CSF2RA and CXCR2 in the respective cell lines. (A) Luciferase-expressing THP-1 cells were counted after 24 h of cultivation. Fold changes were normalized to migration of THP-1 cells in the absence of mono- (empty bars) and co-cultures (black bars). Negative control: No cells (grey bar). Data are based on three biological replicas, each representing three technical replicates. Statistical analysis was performed by using unpaired Student’s t-test (***p<0.001; n.s.: not significant). (B) Experimental setup. (C) Relative linear expression levels of CSF2RA and CXCR2. Tumor cell lines (dark grey), HDFs (light grey), THP-1 (black). Relative expression levels are from Affymetrix GeneChip studies (Exon Chip 1.0). The respective analyzed gene is depicted in the header of each diagram. Statistical analysis was performed on the mean values by one-way ANOVA (*p<0.05, ****p<0.0001).

Interestingly, chemokines CXCL1 and CXCL6 as well as CSF2 have been shown to induce migration of monocytes [86,87,88]. As we have shown that CSF2, CXCL1 and CXCL6 are exclusively induced in FBs co-cultured with tumor cells exhibiting an EMT signature (Fig 5) we next addressed the question of whether THP-1 cell migration can be enhanced by being placed in a triple culture alongside the NSCLC cell line NCI-H157 and HDFs. Therefore, we set up a transwell assay system with luciferase-expressing THP-1 cells in the upper compartment of the transwell device (pore size 3 μm) and the NCI-H157/HDF co-culture in the underlying well. Indeed, in the presence of the co-culture of the invasive cell line (NCI-H157) but not that of the non-invasive cell line (NCI-H1437) a significant induction of migration (>2-fold) through the membrane could be observed for THP-1 (Fig 9A and 9B). However, addition of recombinant CSF2 alone into our co-culture system had neither an effect on migration (S7 Fig) nor on invasion of cell line Calu-1 (S8 Fig). Therefore, it seems likely that the induction of CXCL1 and CXCL6 in FBs may be responsible for the enhanced migration of THP-1 in the triple co-culture. This hypothesis is supported by the fact that the receptor for both CXCL1 and CXCL6, CXCR2, is significantly overexpressed in THP-1 but not by other cell types of the co-cultures (Fig 9C). Interestingly, the receptor for CSF2, CSF2RA, is also significantly overexpressed in THP-1. Clearly, future experiments will be needed to address other roles of CSF2 in the tumor-stroma crosstalk such as suggested contributions in the recruitment of monocytes, macrophages and neutrophils into the tumor vicinity [89,90,91]. Our organotypic 3D models consisting of human tumor cells, stromal FBs and immune cells (or their respective precursors) represent valuable models which will allow to appropriately characterize the mechanisms of crosstalk in those triple cultures.

In summary, utilizing different organotypic co-cultures we were able to identify cytokine fingerprints (“inflammatory signature”) specifically induced upon co-cultivation of FBs and invasive NSCLC tumor cell lines. This signature was induced exclusively in the stromal component but was governed by the invasive phenotype of the associated cancer cells. In the Calu-1 cell line the HGF-MET-ERK1/2-CREB-axis was shown to contribute to the onset of the invasive phenotype with HGF being secreted by FBs. In particular, CSF2 was only induced in co-cultures of several NSCLC invasive cell lines regardless of the partnered FB type. These cultures revealed a clear link between the induction of CSF2 and the EMT signature of the cancer cell line. The canonical NFκB signaling in FBs, but not in tumor cells, was shown to be responsible for the induced and constitutive CSF2 expression. Additional signaling pathways (AP-1, HIF1-α, KLF4, SP-1 and ELK-1) were found to be induced in FBs co-cultured with Calu-1. From our reporter assay data we conclude that only invasive tumor cells are able to stimulate distinct signaling pathways in FBs without being self-activated. In addition, our findings are in good agreement with the fact that we have seen significant NFκB activation in FBs only upon co-cultivation with invasive tumor cells. Therefore, tumor cells might be regarded as the inducers and FBs as the executors. Finally the question remains what makes an invasive tumor cell becoming an inducer and through which signal from the tumor cell CSF2 gets induced in FBs. To address these questions—at least *in silico*—we made use of our transcription profiling study. CSF2 is known to be induced by either IL1B or TNFA [92]. Based on transcription profiling, a two- to three-fold increase of IL1B expression is observed in co-cultures but no changes in the TNFA levels were detected (S9 Fig). Again, induction of IL1B is only seen in co-cultures of FBs with the invasive cell line Calu-1 but not with NCI-H1437 (Fig 7). In addition, the cognate receptor for IL1B, IL1R1, is strongly expressed in all the different FBs but not on the tumor cells (S9 Fig). From this data set we suggest a hypothetical positive activation loop: IL1B has a basal expression in invasive tumor cells leading to induction of IL1R1 in FBs subsequently turning on NFκB signaling in FBs and escalating expression of IL1B in FBs. Finally this leads to a massive expression of CSF2. A model summarizing our findings on the crosstalk between invasive or non-invasive lung tumor cells with FBs is shown in S10 Fig. This hypothesis remains to be proven by functional experiments in the future.

## Supporting Information

S1 Fig Invasive or non-invasive growth behavior of various NSCLC cell lines in a 3D collagen I matrix. Pictures were taken after 72 h of spheroid embedding into collagen I. For details see Materials and Methods. Scale bar = 100 μM.
(TIF)

S2 Fig Three-dimensional co-cultures of Calu-1 cells with primary NF1 and CAF1 cells. Pictures were taken after 24 h of spheroid embedding into collagen I. For details see Materials and Methods.
(TIF)

S3 Fig MET expression level and MET activation in NCI-H1437. (A) Western blot analysis of whole cell lysates from NCI-H1437 mono-cultures prior starved in OPTI-MEM for 6 h and subsequently incubated for 15 min in the respective supernatant (SN). HDF SN: HDF mono-culture SN, NCI-H1437+HDF SN: co-culture derived SN. (B) Relative expression level of MET in NCI-H1437 compared with Calu-1. Data obtained on Affymetrix Exon 1.0 Chip.
(TIF)

S4 Fig Co-cultivation of tumor spheroids of NCI-H157 or NCI-H226 with HDFs does not change the growth phenotype of tumor cells. Invasive cell lines NCI-H157 and NCI-H226 were co-cultivated with human dermal fibroblasts (HDFs) in collagen I for 48 h. Microscope pictures were taken with a brightfield microscope. Scale bar = 100 μm.
(TIF)

S5 Fig Experimental setup for 2D and 3D co-cultures. Illustration of the workflow how to obtain a mixture of RNA, cell lysate or supernatant from mono-cultures of tumor cells (green circles) and FBs (elongated cells in red) as well as from the corresponding co-cultures. A defined number of tumor cells or spheroids were grown for three days with and without an exactly determined cell number of the different FBs. The whole lysates from tumor cell mono-cultures were mixed with FB mono-culture lysates, referred to as mono-culture mix lysate (yellow box), thereby ensuring the same amount of tumor and FB components present as in the co-culture experiments, referred to as co-culture lysate (blue box). Data generated either with the mono-culture mixes or with mono-cultures served as a reference. RNA derived from mono- and co-cultures as well as from mono-culture mixes was analyzed on Affymetrix GeneChips (GeneChip EXON1.0) or used for qPCR. The corresponding cell lysates or cell culture supernatants were subjected to various cytokine and signal transduction array analyses as well as used for ELISA reporter gene assay studies (for details see Materials and Methods).
(TIF)

S6 Fig RT-qPCR for a set of cytokines (CSF2, IL6, IL8 and IL1B) and chemokines (CXCL1 and CXCL6) of total RNA samples derived from mono- and co-cultures in a transwell assay. (A) Co-cultures of NCI-H157 and NCI-H1437 with NF1 and (B) corresponding co-cultures with CAF1. The respective co- (+) or mono- (-) culture is indicated on the X-axis. The analyzed cytokine/chemokine is indicated in the header of each graph. Expression values are shown in arbitrary units (AU) and have been normalized to beta-2 microglobulin (B2M) mRNA copies. Statistical analysis was performed on the mean values by unpaired comparison of mono-cultured NF1 or CAF1 and co-cultured NF1 or CAF1 RNA samples by using Student’s t-test (*p<0.05, **p<0.01, ***p<0.001; n.s.: not significant).
(TIF)

S7 Fig Transwell migration assay of THP-1 cells with recombinant CSF2. CSF2 was added into the bottom chamber. Luciferase-expressing THP-1 cells were counted after 24 h of cultivation with recombinant CSF2. Fold changes are normalized to migration of THP-1 cells in the absence of CSF2 (0 ng/ml). Data are based on three biological replicas, each representing three technical replicates. Statistical analysis was performed by using unpaired Student’s t-test (*p<0.05; n.s.: not significant).
(TIF)

S8 Fig Calu-1 invasion assay in the presence of various concentrations of CSF2. Calu-1 spheroids were embedded into collagen I and incubated for 48 h with 0, 50 and 250 ng/ml of recombinant human CSF2 (R&D).
(TIF)

S9 Fig IL1B, TNFA and IL1R1 transcription profiles of mono- and co-cultures. Data are based on triplicates. Relative expression levels are shown on the Y-axis in arbitrary units (AU). The bold centerline indicates the median; the box represents the interquartile range (IQR). Whiskers extend to 1.5 times the IQR.
(TIF)

S10 Fig Molecular model of tumor-stroma interactions between invasive (A) or non-invasive (B) lung tumor cells with fibroblasts. HGF is secreted by FBs and leads to the activation of MET in the tumor cells. The MAPK pathway in invasive NSCLC cells is turned on and leads to CREB phosphorylation. Thereby collective invasion of invasive tumor cells switches to aggressive scattering of single cells into the extracellular matrix. Based on the EMT signature score invasive tumor cells efficiently activate NFκB and AP-1 signaling in FBs whereas FBs co-cultured with non-invasive tumor cells exhibit only a residual NFκB and no AP-1 activation. Activation of NFκB and AP-1 target sites in FBs co-cultured with invasive tumor cells further leads to expression, translation and release of cytokines and chemokines, such as IL1B, CSF2, CXCL1, IL6, CXCL6 and IL8. Subsequently, IL1B acts in an autocrine fashion through binding to the activating IL1 receptor (IL1R1) on FBs to ensure continuous NFκB signaling activation. In contrast, a co-culture with non-invasive tumor cells leads to the secretion of a smaller subset of cytokines such as CXCL1, IL6 and CXCL6. The induced increase in migration of a monocytic cell (e.g. THP-1) in a triple culture might be due to the secreted cytokine cocktail and the corresponding receptors (IL6R, CXCR2 and CSF2RA) found to be exclusively expressed on the monocytic cell line THP-1. However, the underlying molecular mechanism of how invasive cancer cells force FBs to produce those cytokines remains to be determined (dotted red arrow).
(TIF)

S1 Table Cell Line Description. Cell and histology type, origin and source of the respective cell lines are indicated. NF1/CAF1 and NF2/CAF2 are patient-matched pairs.
(XLSX)

S2 Table Genes/ gene products reported to either interact with NFκB or are direct/ indirect target genes of NFκB. Unless indicated otherwise, the list is based on the following data base and cited publications:

http://www.bu.edu/nf-kb/gene-resources/target-genes/ (Boston List NFκB Target Genes).Takase *et al*., Clin Exp Nephrol (2008) 12: 181–188.Shelest *et al*., In Silico Biology (2003) 3: 71–79.Liu *et al*., Genome Res. (2003) 13: 654–661.Naamane *et al*., BMC Bioinformatics (2007) 8: 55.
(XLSX)

S3 Table Upregulated genes from the top ranked canonical pathways identified in all four different co-cultures of each tumor cell line. Selection parameters are: FC<-1.5 or FC>1.5 and p<0.01. Genes linked to NFκB are indicated (see S2 Table). Genes are extracted from the Ingenuity Canonical Pathway Analysis (see also Table 2).
(XLSX)

S4 Table Statistical analysis of ELISA and antibody array data. Statistical analysis was performed on the mean values by unpaired comparison using Student’s t-test. p-values are shown for non-invasive NCI-H1437 co-cultures and invasive Calu-1 co-cultures.
(XLSX)
