# Supplementary figures and images for: High EMT Signature Score of Invasive Non-Small Cell Lung Cancer (NSCLC) Cells Correlates with NFκB Driven Colony-Stimulating Factor 2 (CSF2/GM-CSF) Secretion by Neighboring Stromal Fibroblasts
Source: PLoS One. 2015 Apr 28;10(4):e0124283. doi: 10.1371/journal.pone.0124283 (PMC4412534; doi:10.1371/journal.pone.0124283)

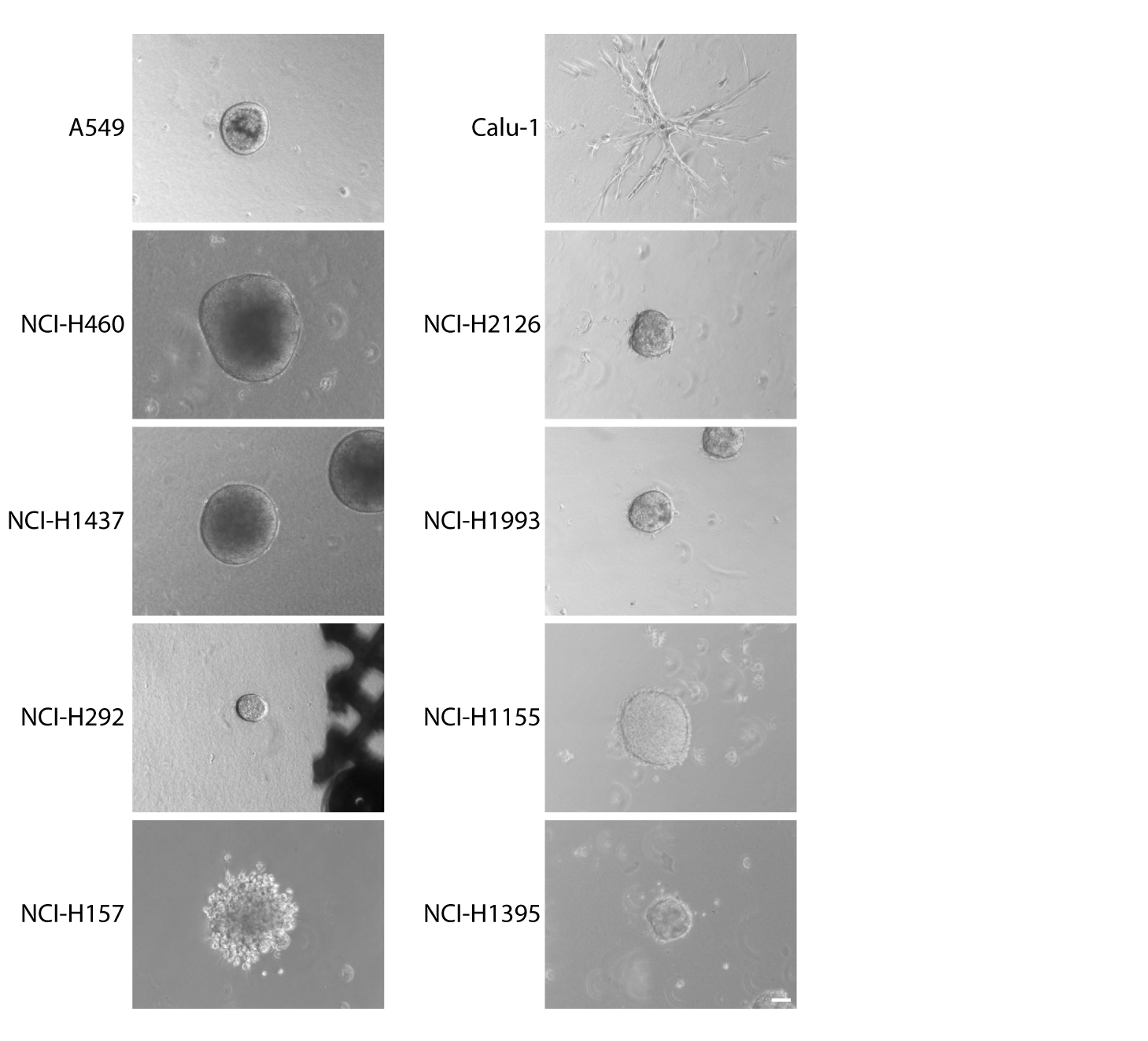

Supplement: S1 Fig — Pictures were taken after 72 h of spheroid embedding into collagen I. For details see Materials and Methods. Scale bar = 100 μM. (TIF) [file pone.0124283.s001.tif]

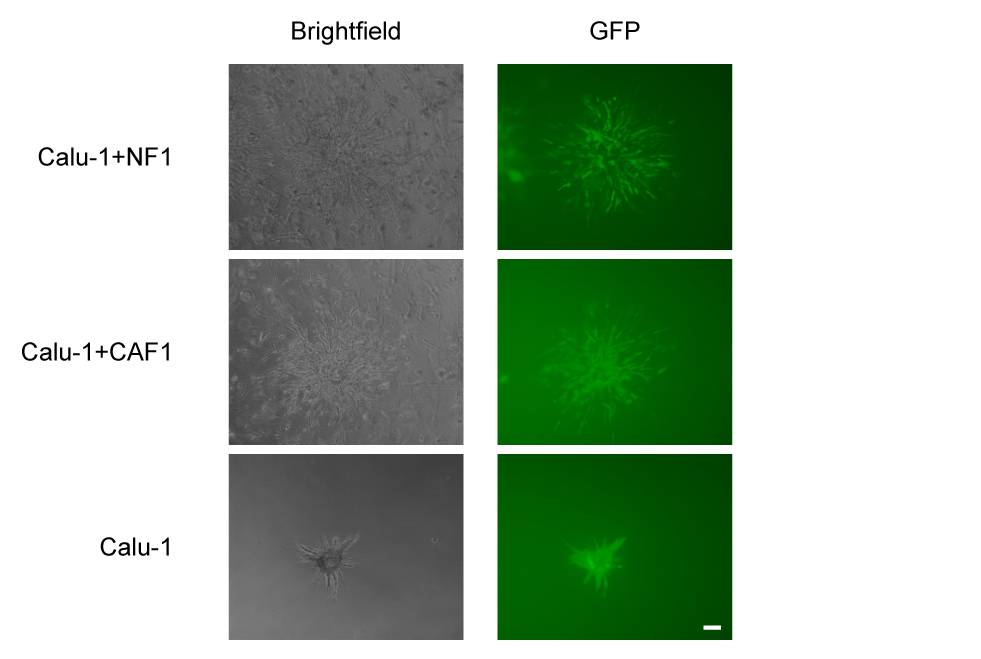

Supplement: S2 Fig — Pictures were taken after 24 h of spheroid embedding into collagen I. For details see Materials and Methods. (TIF) [file pone.0124283.s002.tif]

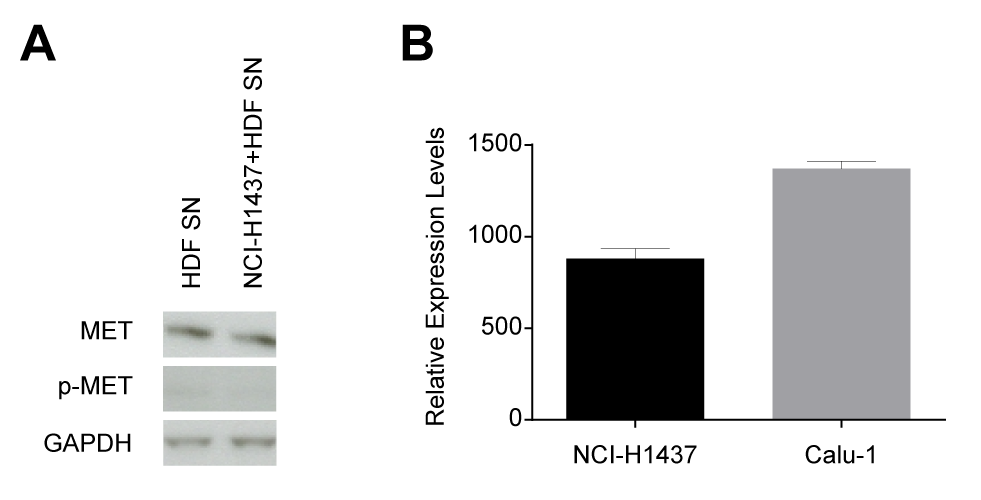

Supplement: S3 Fig — (A) Western blot analysis of whole cell lysates from NCI-H1437 mono-cultures prior starved in OPTI-MEM for 6 h and subsequently incubated for 15 min in the respective supernatant (SN). HDF SN: HDF mono-culture SN, NCI-H1437+HDF SN: co-culture derived SN. (B) Relative expression level of MET in NCI-H1437 compared with Calu-1. Data obtained on Affymetrix Exon 1.0 Chip. (TIF) [file pone.0124283.s003.tif]

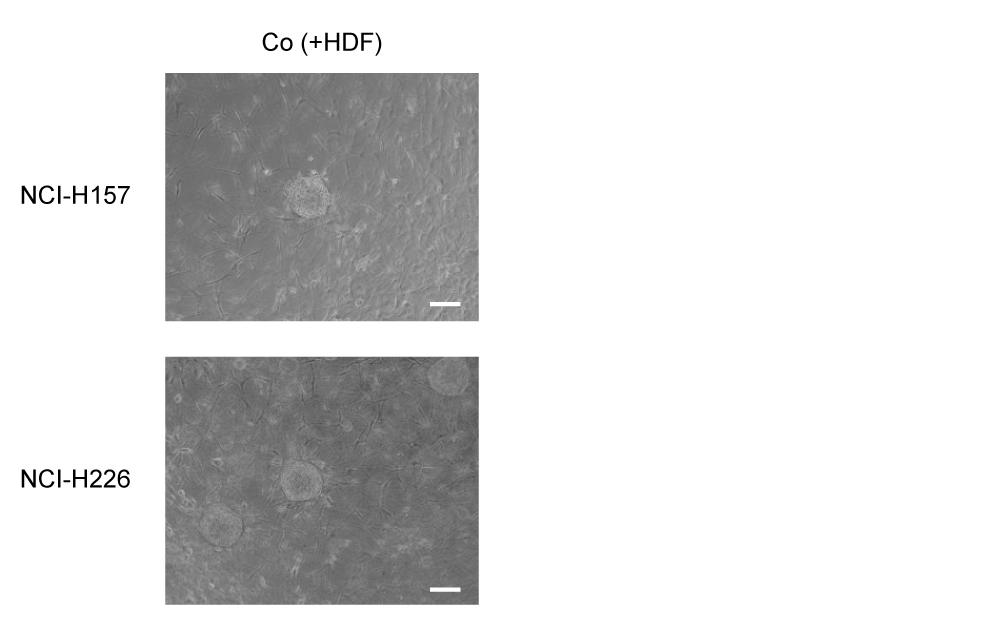

Supplement: S4 Fig — Invasive cell lines NCI-H157 and NCI-H226 were co-cultivated with human dermal fibroblasts (HDFs) in collagen I for 48 h. Microscope pictures were taken with a brightfield microscope. Scale bar = 100 μm. (TIF) [file pone.0124283.s004.tif]

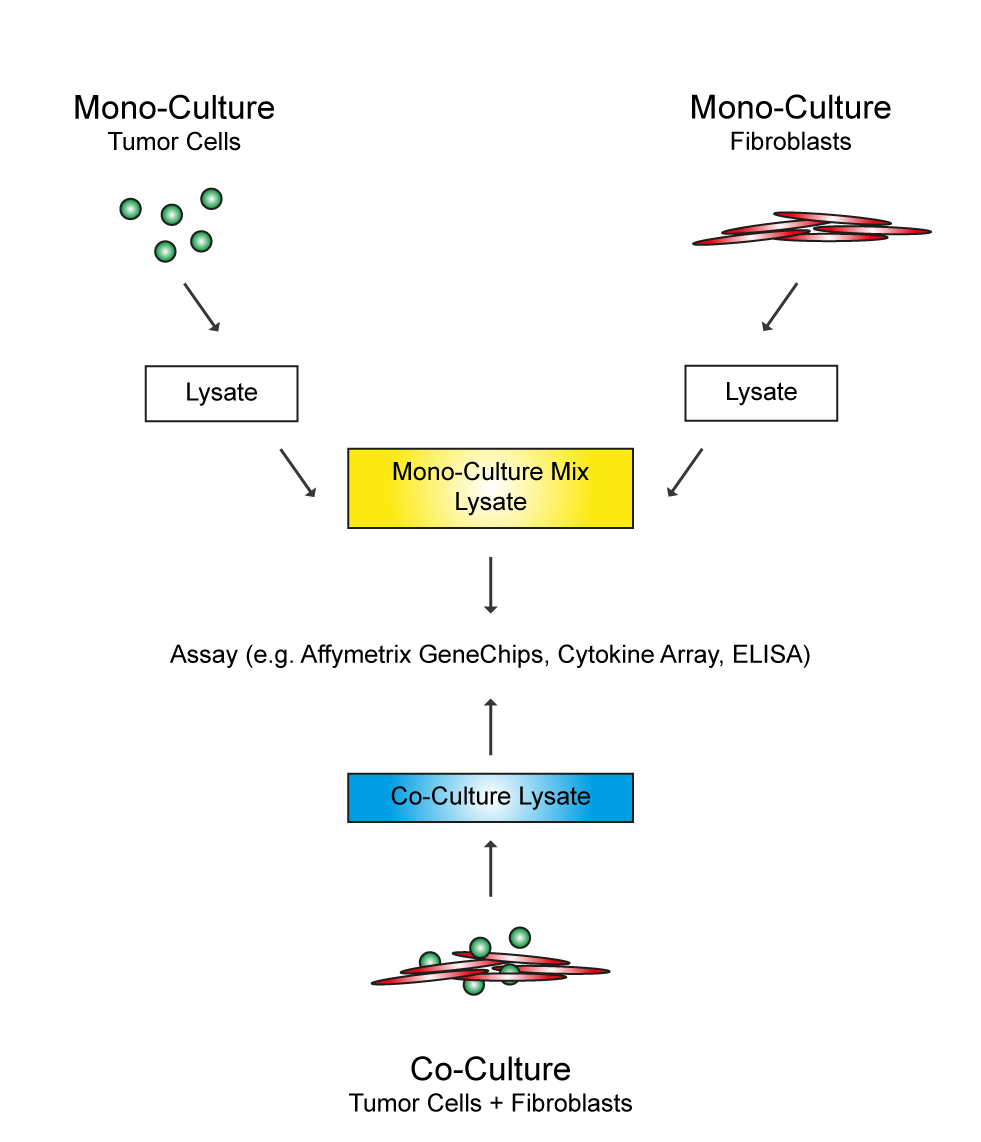

Supplement: S5 Fig — Illustration of the workflow how to obtain a mixture of RNA, cell lysate or supernatant from mono-cultures of tumor cells (green circles) and FBs (elongated cells in red) as well as from the corresponding co-cultures. A defined number of tumor cells or spheroids were grown for three days with and without an exactly determined cell number of the different FBs. The whole lysates from tumor cell mono-cultures were mixed with FB mono-culture lysates, referred to as mono-culture mix lysate (yellow box), thereby ensuring the same amount of tumor and FB components present as in the co-culture experiments, referred to as co-culture lysate (blue box). Data generated either with the mono-culture mixes or with mono-cultures served as a reference. RNA derived from mono- and co-cultures as well as from mono-culture mixes was analyzed on Affymetrix GeneChips (GeneChip EXON1.0) or used for qPCR. The corresponding cell lysates or cell culture supernatants were subjected to various cytokine and signal transduction array analyses as well as used for ELISA reporter gene assay studies (for details see Materials and Methods). (TIF) [file pone.0124283.s005.tif]

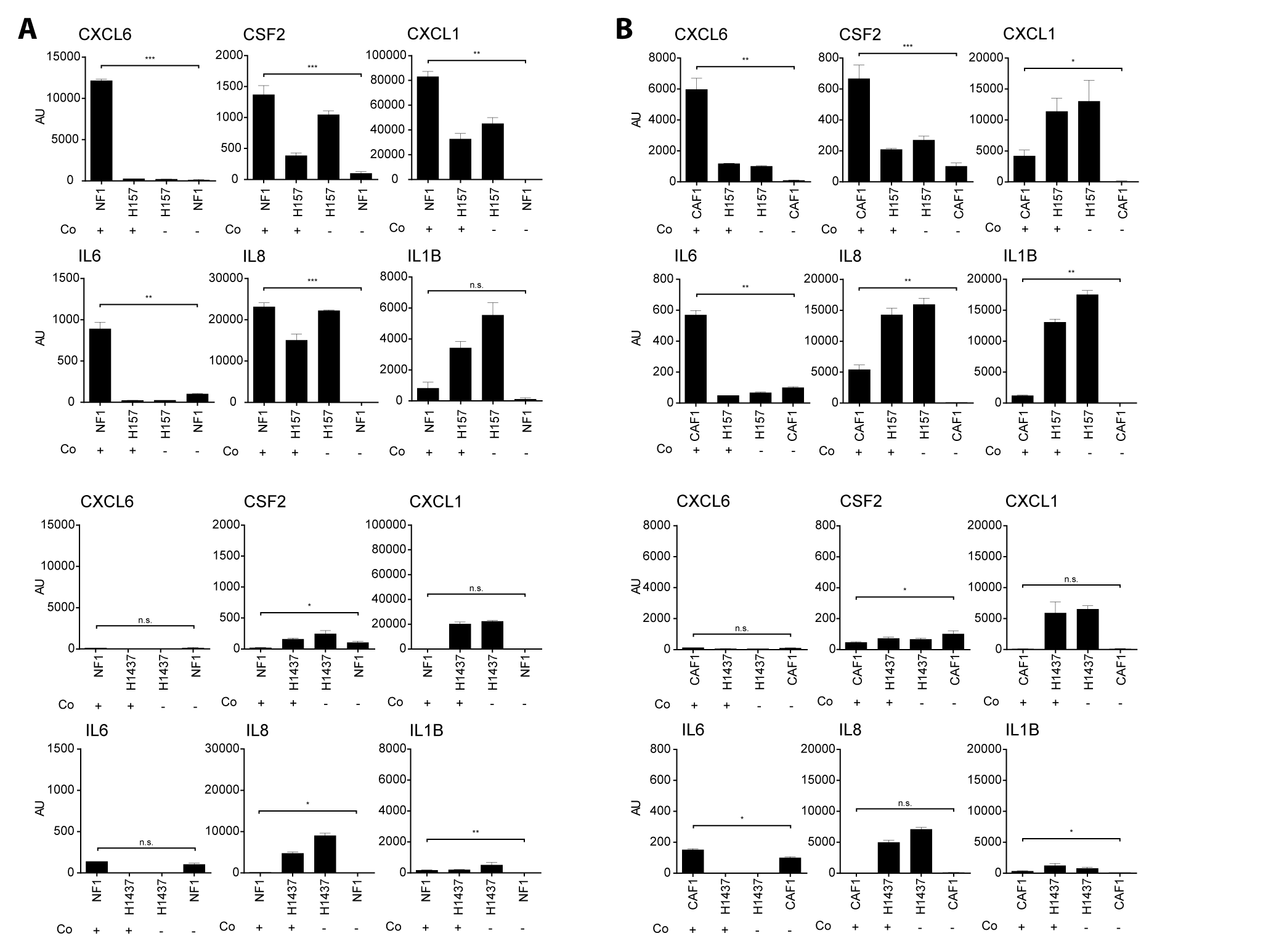

Supplement: S6 Fig — (A) Co-cultures of NCI-H157 and NCI-H1437 with NF1 and (B) corresponding co-cultures with CAF1. The respective co- (+) or mono- (-) culture is indicated on the X-axis. The analyzed cytokine/chemokine is indicated in the header of each graph. Expression values are shown in arbitrary units (AU) and have been normalized to beta-2 microglobulin (B2M) mRNA copies. Statistical analysis was performed on the mean values by unpaired comparison of mono-cultured NF1 or CAF1 and co-cultured NF1 or CAF1 RNA samples by using Student’s t-test (*p<0.05, **p<0.01, ***p<0.001; n.s.: not significant). (TIF) [file pone.0124283.s006.tif]

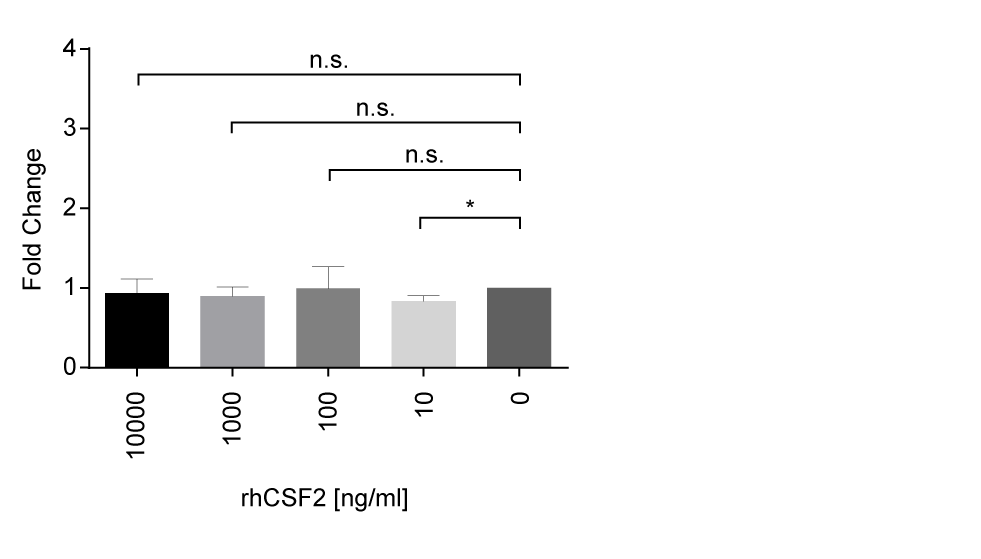

Supplement: S7 Fig — CSF2 was added into the bottom chamber. Luciferase-expressing THP-1 cells were counted after 24 h of cultivation with recombinant CSF2. Fold changes are normalized to migration of THP-1 cells in the absence of CSF2 (0 ng/ml). Data are based on three biological replicas, each representing three technical replicates. Statistical analysis was performed by using unpaired Student’s t-test (*p<0.05; n.s.: not significant). (TIF) [file pone.0124283.s007.tif]

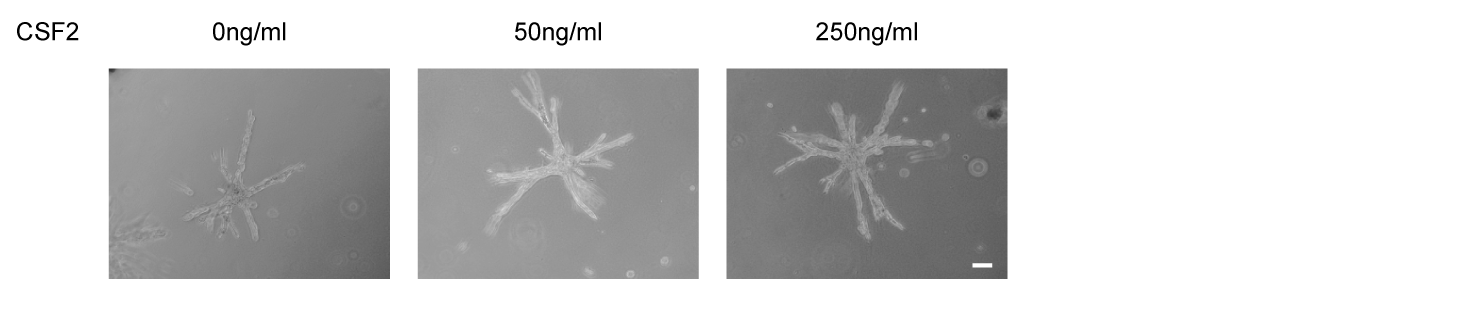

Supplement: S8 Fig — Calu-1 spheroids were embedded into collagen I and incubated for 48 h with 0, 50 and 250 ng/ml of recombinant human CSF2 (R&D). (TIF) [file pone.0124283.s008.tif]

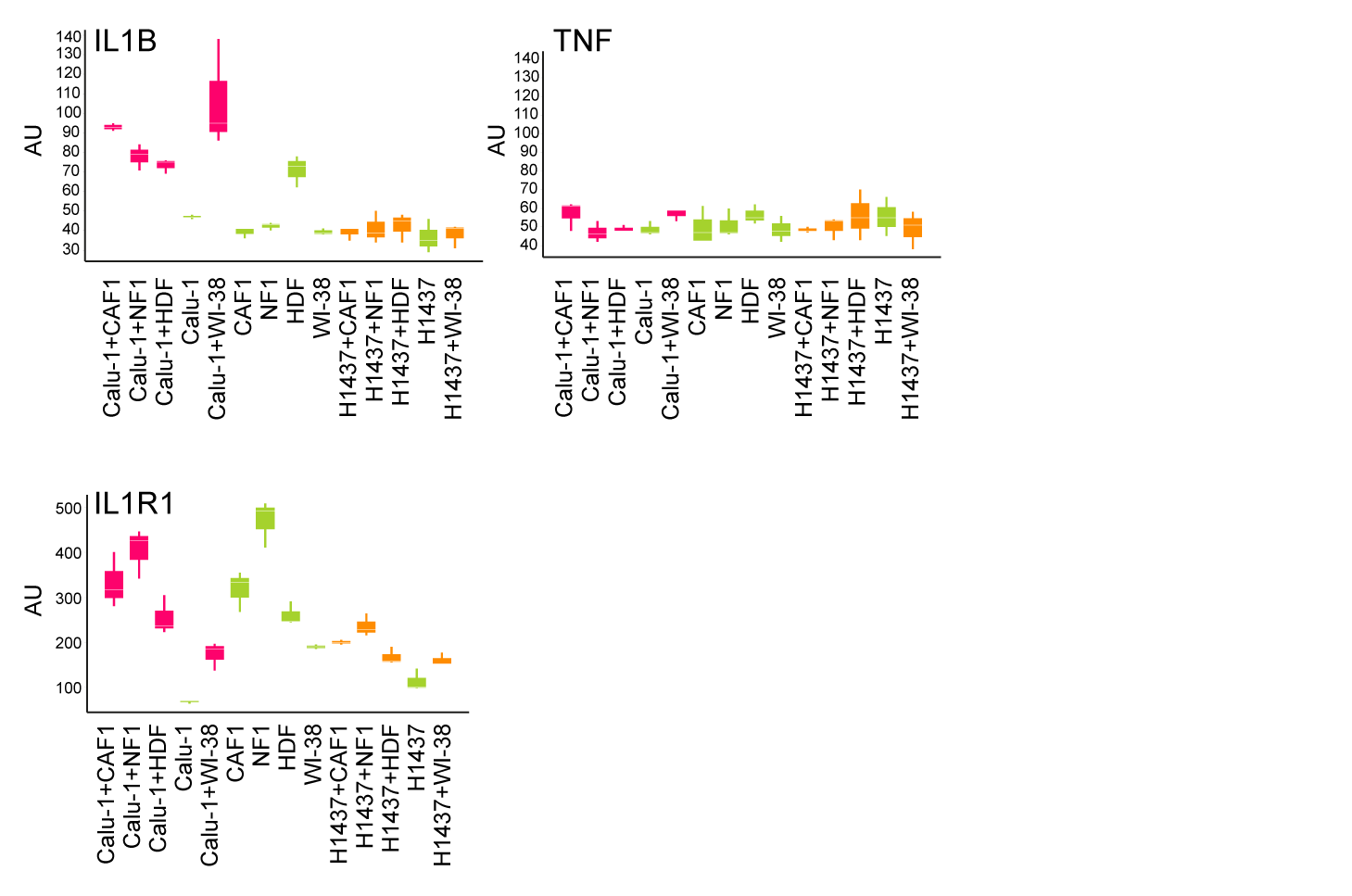

Supplement: S9 Fig — Data are based on triplicates. Relative expression levels are shown on the Y-axis in arbitrary units (AU). The bold centerline indicates the median; the box represents the interquartile range (IQR). Whiskers extend to 1.5 times the IQR. (TIF) [file pone.0124283.s009.tif]

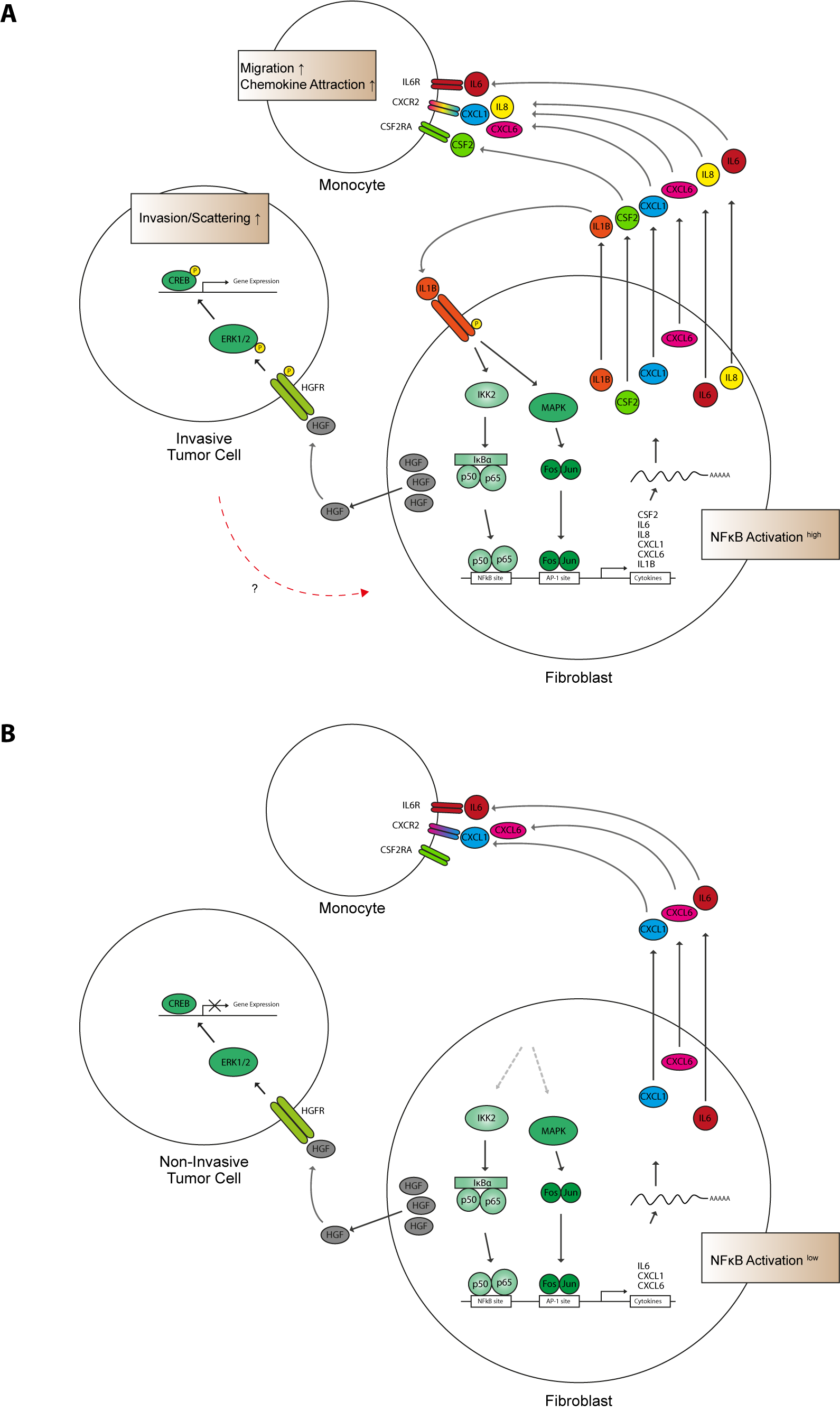

Supplement: S10 Fig — HGF is secreted by FBs and leads to the activation of MET in the tumor cells. The MAPK pathway in invasive NSCLC cells is turned on and leads to CREB phosphorylation. Thereby collective invasion of invasive tumor cells switches to aggressive scattering of single cells into the extracellular matrix. Based on the EMT signature score invasive tumor cells efficiently activate NFκB and AP-1 signaling in FBs whereas FBs co-cultured with non-invasive tumor cells exhibit only a residual NFκB and no AP-1 activation. Activation of NFκB and AP-1 target sites in FBs co-cultured with invasive tumor cells further leads to expression, translation and release of cytokines and chemokines, such as IL1B, CSF2, CXCL1, IL6, CXCL6 and IL8. Subsequently, IL1B acts in an autocrine fashion through binding to the activating IL1 receptor (IL1R1) on FBs to ensure continuous NFκB signaling activation. In contrast, a co-culture with non-invasive tumor cells leads to the secretion of a smaller subset of cytokines such as CXCL1, IL6 and CXCL6. The induced increase in migration of a monocytic cell (e.g. THP-1) in a triple culture might be due to the secreted cytokine cocktail and the corresponding receptors (IL6R, CXCR2 and CSF2RA) found to be exclusively expressed on the monocytic cell line THP-1. However, the underlying molecular mechanism of how invasive cancer cells force FBs to produce those cytokines remains to be determined (dotted red arrow). (TIF) [file pone.0124283.s010.tif]
